# Mapping human pluripotent stem cell differentiation pathways using high throughput single-cell RNA-sequencing

**DOI:** 10.1186/s13059-018-1426-0

**Published:** 2018-04-05

**Authors:** Xiaoping Han, Haide Chen, Daosheng Huang, Huidong Chen, Lijiang Fei, Chen Cheng, He Huang, Guo-Cheng Yuan, Guoji Guo

**Affiliations:** 1 Center for Stem Cell and Regenerative Medicine, Zhejiang University School of Medicine, Hangzhou, 310058 China; 2 Institute of Hematology, The 1st Affiliated Hospital, Zhejiang University School of Medicine, Hangzhou, 310003 China; 3 Dr. Li Dak Sum & Yip Yio Chin Center for Stem Cell and Regenerative Medicine, Zhejiang Provincial Key Lab for Tissue Engineering and Regenerative Medicine, Hangzhou, 310058 China; 4 Department of Biostatistics and Computational Biology, Dana-Farber Cancer Institute, Harvard Chan School of Public Health, Boston, MA 02115 USA; 5 College of Animal Science, Zhejiang University, Hangzhou, 310058 China; 6 Department of Computer Science and Technology, Tongji University, Shanghai, 201804 China; 7 College of Life Sciences, Zhejiang University, Hangzhou, 310058 China; 8 Stem Cell Institute, Zhejiang University, Hangzhou, 310058 China

**Keywords:** Single-cell RNA-sequencing, Primed human pluripotent stem cell, Embryoid body, Naïve human pluripotent stem cell

## Abstract

**Background:**

Human pluripotent stem cells (hPSCs) provide powerful models for studying cellular differentiations and unlimited sources of cells for regenerative medicine. However, a comprehensive single-cell level differentiation roadmap for hPSCs has not been achieved.

**Results:**

We use high throughput single-cell RNA-sequencing (scRNA-seq), based on optimized microfluidic circuits, to profile early differentiation lineages in the human embryoid body system. We present a cellular-state landscape for hPSC early differentiation that covers multiple cellular lineages, including neural, muscle, endothelial, stromal, liver, and epithelial cells. Through pseudotime analysis, we construct the developmental trajectories of these progenitor cells and reveal the gene expression dynamics in the process of cell differentiation. We further reprogram primed H9 cells into naïve-like H9 cells to study the cellular-state transition process. We find that genes related to hemogenic endothelium development are enriched in naïve-like H9. Functionally, naïve-like H9 show higher potency for differentiation into hematopoietic lineages than primed cells.

**Conclusions:**

Our single-cell analysis reveals the cellular-state landscape of hPSC early differentiation, offering new insights that can be harnessed for optimization of differentiation protocols.

**Electronic supplementary material:**

The online version of this article (10.1186/s13059-018-1426-0) contains supplementary material, which is available to authorized users.

## Background

Thomson et al. derived human pluripotent stem cells (hPSCs) from human blastocysts for the first time in 1998 [1]. hPSCs have the capacity of self-renewal and multilineage differentiation both in vitro and in vivo. These features of hPSCs have provided remarkable promise in developmental biology and regenerative medicine [2]. hPSCs can be used to generate diverse cell-types from all three germ layers using different differentiation protocols [3–7]. However, most existing protocols suffer from low efficiency and functional deficiency.

**Fig. 1:**
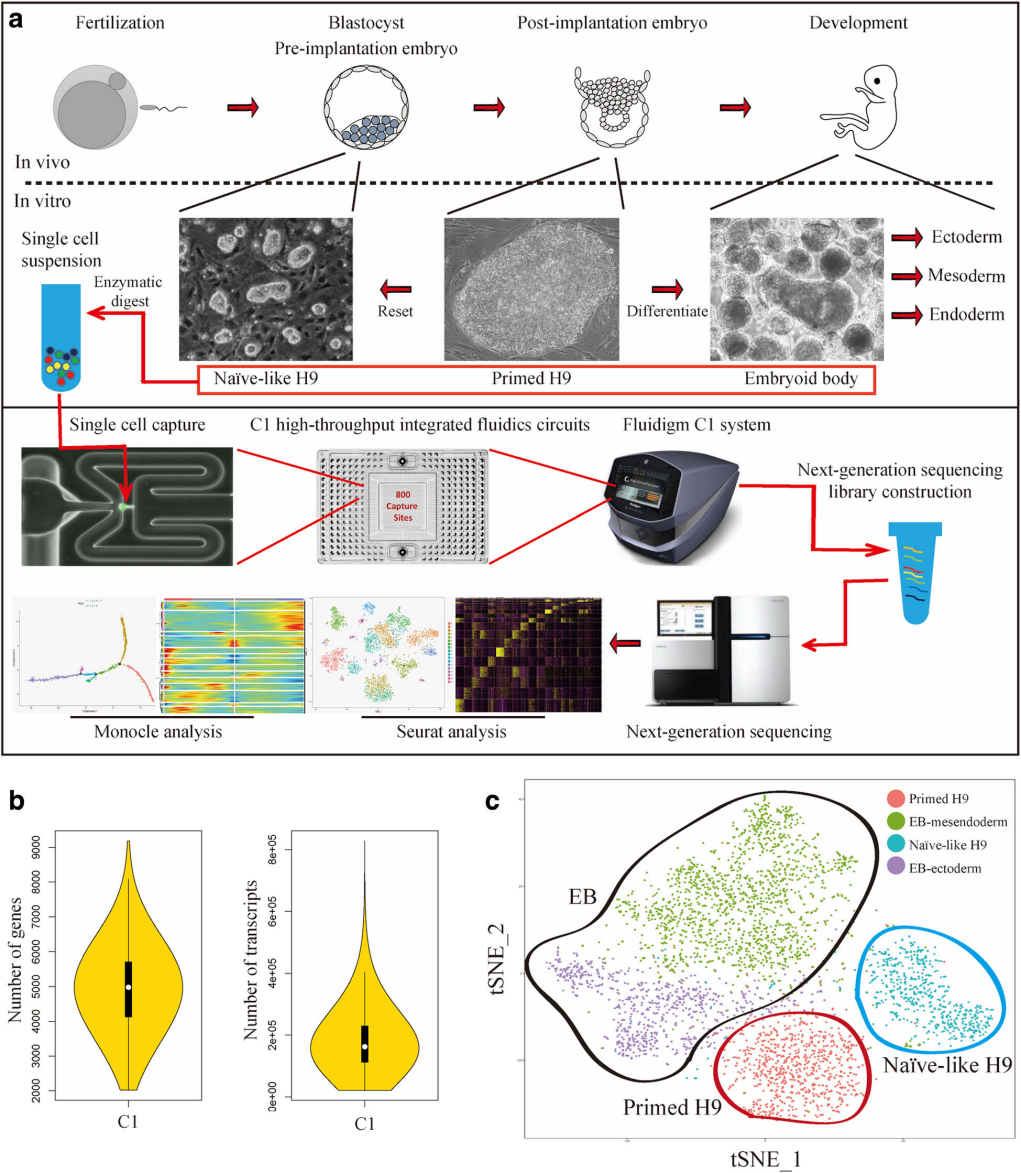
Overview of scRNA-seq analysis on hPSC early differentiation. **a** *Process flow diagram* of scRNA-seq analysis on hPSC early differentiation. Single-cell samples of Naïve-like H9, Primed H9, and EBs were prepared by Fluidigm C1 system with HT IFCs for sequencing. Data analysis was performed using Seurat and Monocle. **b** *Violin plots* show the distribution of transcripts and genes detected per cell. **c** *t-SNE plot* of single-cell samples profiled. Naïve-like H9 cluster (*blue circle*), EB clusters (*black circle*), Primed H9 cluster (*red circle*)

In vivo, fertilized mammalian eggs undergo multiple cleavage divisions and form blastocysts (Fig. 1a). The pre-implantation mouse epiblasts obtained from blastocysts have the ground-state naïve pluripotency that can be recapitulated in vitro in the form of embryonic stem cells (ESCs) [8, 9]. Soon after implantation, epiblasts become primed for lineage specification. In vitro, the counterparts of primed epiblasts are termed epiblast stem cells (EpiSCs), which are functionally and morphologically distinct from ESCs. These two states of pluripotent stem cells (i.e. ESCs and EpiSCs) are interchangeable under specific conditions [9]. The study of this cellular-state transition process will contribute to the understanding of early development from pre-implantation epiblasts to post-implantation epiblasts. Conventional hPSCs are considered as the primed state with the molecular and functional identity of post-implantation lineage-primed epiblasts. Several groups have established the naïve hPSCs, which share several molecular features and functional characteristics with naïve mPSCs and pre-implantation epiblasts [10–16]. Following lineage specification, primed epiblasts develop into embryonic ectoderm and primitive streak, which further develop into embryonic mesoderm and endoderm. These three embryonic germ layers develop into all embryonic tissues. Under proper in vitro culture conditions, hPSCs can undergo spontaneous differentiation and form a three-dimensional (3D) structure called embryoid body (EB), which contains cells from all three germ layers [17, 18]. The EB differentiation system is a widely used model to study the early differentiation of various lineage-specific progenitors, including cardiac muscle [19], blood [20], liver [21], and neuron [22], etc. hPSCs (both primed and naïve) and EBs are powerful models to simulate early developmental process in vitro, from pre-implantation epiblasts to lineage-committed progenitors.

hPSC differentiation is a complex process. Flow cytometry and immunostaining have been used to define cell types in hPSC differentiation cultures. However, these methods are limited by the number of fluorescent probes that can be used at the same time; the heterogeneity of the hPSC differentiation process cannot be fully resolved. Single-cell RNA-sequencing (scRNA-seq), first released in 2009 [23], has provided a promising alternative. During the past few years, the technology has been vastly improved by the development of numerous innovative approaches [24, 25], including C1 (SMARTer) [26], SMART-seq2 [27], CEL-seq [28], Drop-seq [29], InDrop [30], 10X Genomics [31], etc. To date, single-cell technology has been used to study cellular heterogeneity in a wide range of systems [24], including the hierarchy of tumor cells [32, 33], tissue and organs [34–37], developing embryos [38, 39] and in vitro differentiation systems [40–42].

We use the upgraded Fluidigm C1 system with optimized high-throughput integrated fluidics circuits (HT IFCs) to construct the early differentiation trajectories of various lineage-specific progenitors derived from hPSCs and to reveal the interaction between these precursor cells in EB differentiation system. We find key TFs and signaling pathways that direct the differentiation process. We show that liver may be involved in regulating the differentiation of other tissue cells through cell–cell interactions. We also use the C1 scRNA-seq platform to study the primed-to-naïve transition process and to reveal the differences in gene expression profiles between Primed and Naïve-like H9. Combined with the analysis of EB differentiation, genes related to hemogenic endothelium development and MAPK-ERK1/2 signaling pathway are enriched in Naïve-like H9 but not in Primed H9. Functionally, Naïve-like H9 show the differentiation bias to endothelial-hematopoietic lineages. Taken together, we construct a comprehensive single-cell level differentiation roadmap for hPSCs and offer new insights into early embryonic lineages that can guide the establishment and optimization of more sophisticated differentiation system.

## Results

### scRNA-seq analysis of hPSCs and EBs

In order to systematically map hPSCs early differentiation pathways, Naïve-like H9, Primed H9, and EBs were prepared as single-cell samples for sequencing using Fluidigm C1 system with HT IFCs (Fig. 1a). This system can be used to analyze up to 800 cells at a time and detect an average of 5000 genes per cell. A major advantage of this technology is the balance of throughput and resolution. After sequencing and data processing, we got high-quality transcriptomic data from 4822 single cells, including 2636 EB samples (683 day 4 EBs and 1953 day 8 EBs), 1491 Naïve-like H9 samples, and 695 Primed H9 samples. The scRNA-seq data had high read depth, which can map to 5000 genes for most of the single-cell samples (Fig. 1b and Additional file 1: Figure S1a); and Naïve-like H9 datasets show weak batch effect of Fluidigm C1 system (Additional file 1: Figure S1b). The random differentiation of EBs causes the batch effect (Additional file 1: Figure S1c). We used Seurat to perform principal component analysis (PCA) and t-distributed stochastic neighbor embedding (t-SNE) analysis [43]. Seurat divided our samples into four main clusters, including two EB clusters (EB-ectodetm and EB-mesendoderm), one Primed H9 cluster, and one Naïve-like H9 cluster (Fig. 1c). hPSCs (i.e. Naïve-like and Primed H9) have relative homogeneity. EB cells show significant heterogeneity, which indicated well spontaneous differentiation of hPSCs and provided a variety of samples for Monocle pseudotime analysis [44]. To reveal the gene expression dynamics and key regulators of hPSC early differentiation, we used Seurat and Monocle to analyze these data.

### Mapping cellular landscape for early embryonic lineages

**Fig. 2:**
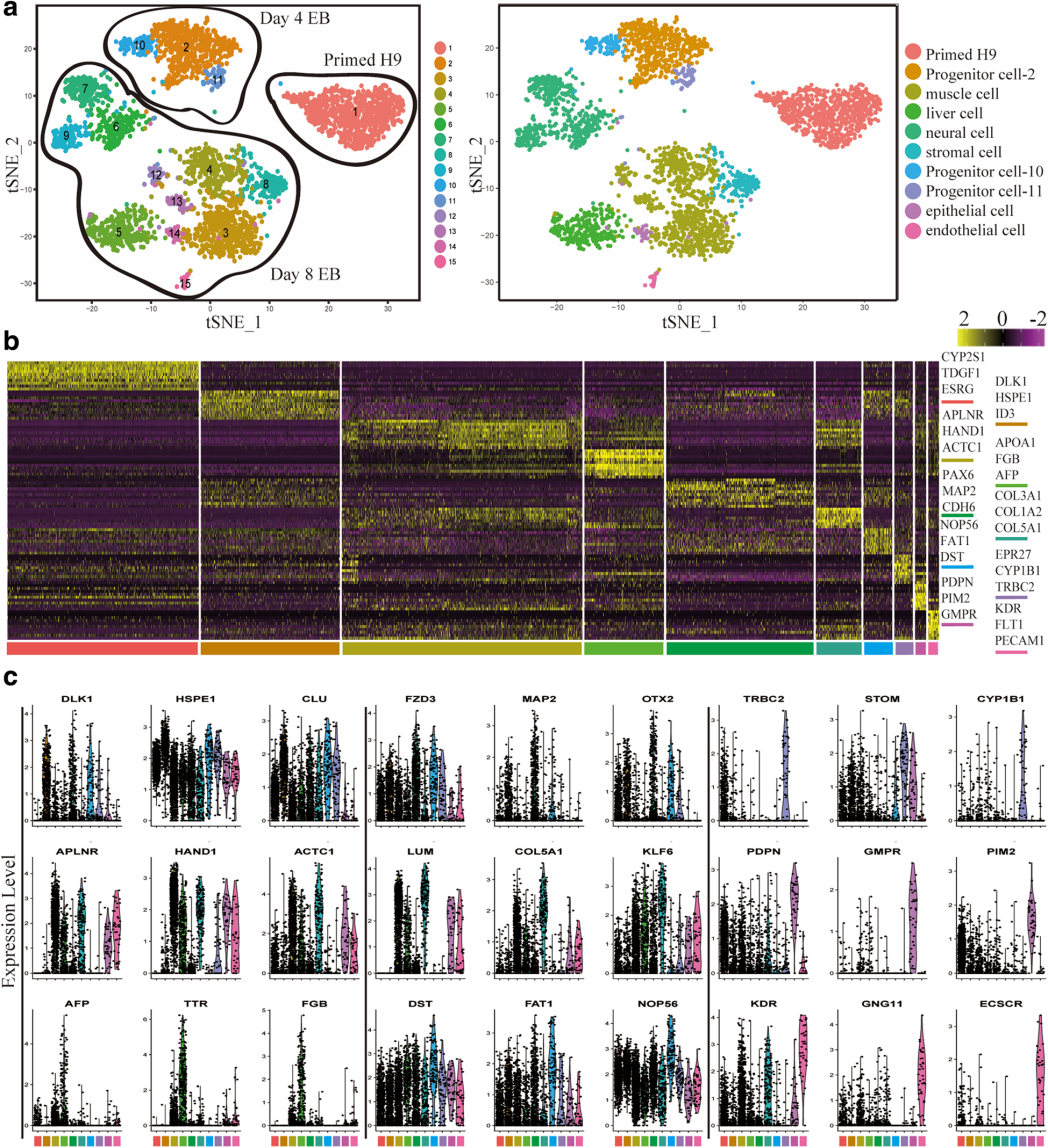
scRNA-seq analysis reveals lineage progenitors in EBs. **a** *t-SNE plots* of Primed H9 and EBs (day 4 EBs and day 8 EBs). We defined three progenitor clusters in day 4 EBs, including progenitor cell-2, progenitor cell-10, and progenitor cell-11. We defined six progenitor clusters in day 8 EBs, including muscle cell, liver cell, neural cell, stromal cell, epithelial cell, and endothelial cell. **b** *Heatmap* shows the expression pattern of top 15 differential genes in each progenitor cell. Differential genes of each cell type are listed in Additional file 4: Table S3. **c** *Violin plots* show the expression level distributions of marker genes across cell types. Cell types are represented by different colors in (**a**), (**b**), and (**c**)

Spontaneous differentiation of EBs exhibit heterogeneous patterns of differentiated cell types (Fig. 1c). We extracted single cells from EBs and Primed H9 for further analysis (Fig. 2a). According to the expression of differential genes, day 4 EBs were divided into three clusters, including progenitor cell-2, progenitor cell-10, and progenitor cell-11. Day 4 EBs have weak heterogeneity. Progenitor cell-2 does not highly express lineage-related genes; progenitor cell-10 may be related with neural cell differentiation; progenitor cell-11 may be related with mesendoderm differentiation (Fig. 2b and c). We defined 11 clusters as different progenitor cells in day 8 EBs. We identified six major types of progenitor cells with distinct gene expression patterns, including muscle cells (cluster 3, 4, 12, 13), stromal cells (cluster 8), endothelial cells (cluster 15), neural cells (cluster 6, 7, 9), epithelial cells (cluster 14), and liver cells (cluster 5) (Fig. 2a and b). Clusters 3, 4, 12, and 13 are associated with high expression of muscle progenitor cell markers such as *HAND1*, *APLNR*, and *ACTC1* [45], and therefore these clusters are annotated as muscle cells (Fig. 2). Cluster 8 is annotated as stromal cells for the expression of *LUM*, *KLF6*, and *COL5A1* [46]. Though muscle cell and stromal cell clusters exhibit shared gene expression profiles, collagen genes (e.g. *COL3A1*, *COL5A1*, *COL5A2*, *COL1A1*, and *COL1A2*) are enriched in stromal cell cluster (Fig. 2b and c) [46]. Cluster 15 is annotated as endothelial cells for the high expression of *KDR*, *GNG11*, and *ECSCR* (Fig. 2b and c) [47]. Clusters 6, 7, and 9 are annotated as neural cells for the high expression of *OTX2*, *PTN*, and *FZD3* (Fig. 2b and c), which are important for the development of neural system [48–50]. Cluster 14 is annotated as epithelial cells for the high expression of *PDPN*, *TFAP2C*, and *DMD* [36, 51]. Cluster 5 is annotated as liver cells for the high expression of *AFP*, *TTR*, and *FGB* [52–54]. We also found specific surface markers to separate these progenitor cells from EBs, such as CD34 and PROCR (CD201), which can enrich endothelial cells from EBs (Additional file 1: Figure S2a and S2b). As a further validation, we found that genes specifically expressed in each cell type were enriched for the expected Gene Ontology (GO) terms (Additional file 1: Figure S2c) [55]. For example, genes that are specifically expressed in muscle cell cluster are significantly enriched for skeletal system development (*p =* 7.06E-07); specific genes of neural cell cluster are significantly enriched for positive regulation of neuroblast proliferation (*p =* 1.86E-04) and neuronal stem cell population maintenance (*p =* 2.17E-04); specific genes of liver cell cluster are significantly enriched for very-low-density lipoprotein particle (*p =* 4.29E-08) and lipoprotein metabolic process (*p =* 5.15E-08); specific genes of endothelial cell cluster are significantly enriched for angiogenesis (*p =* 1.90E-12), positive regulation of endothelial cell proliferation (*p =* 4.40E-05), and hemopoiesis (*p =* 0.0045). These analyses strongly indicate that our cell-type assignments are accurate.

**Fig. 3:**
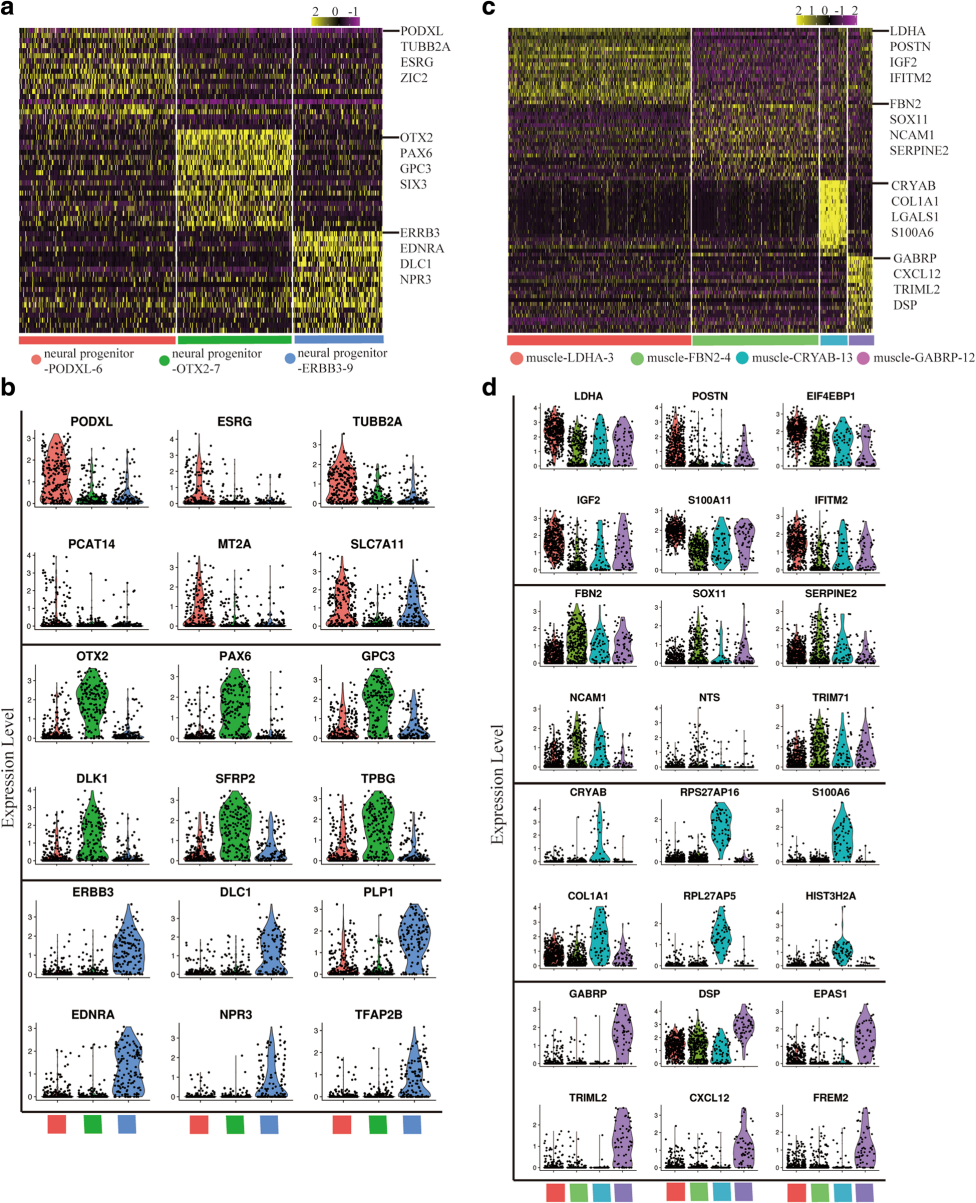
Sub-clusters of neural and muscle progenitors. **a**, **c** *Heatmaps* show the differential gene expression pattern of each sub-cluster from neural cell cluster (**a**) and muscle cell cluster (**c**). Top 20 differential genes of each sub-cluster are shown. Differential genes of each sub-cluster are listed in Additional file 5: Table S4. **b**, **d** *Violin plots* show the expression distributions of specific marker genes across sub-clusters: neural sub-clusters (**b**) and muscle sub-clusters (**d**). Cell types are represented by different *colors*

Both cluster neural cell and muscle cell consist of several sub-clusters. According to the specific gene expression pattern, neural cell cluster was further divided into three subsets, including neural progenitor-PODXL-6, neural progenitor-OTX2–7, and neural progenitor-ERBB3–9 (Fig. 3a and b and Additional file 1: Figure S3a–d). Sub-clusters of neural cells are different types of cells. Neural progenitor-PODXL-6 may be related with cerebral cortex development, because of the enriched expression of *PODXL* [56], *DRAXIN* [57, 58], and *TUBB2A* [59]. Neural progenitor-OTX2–7 is enriched for *RAX*, *SIX3*, and *OTX2*, which are highly expressed in retinal-pigmented epithelium (RPE) [60]. Genes related with forebrain development are enriched in neural progenitor-OTX2–7 (Additional file 1: Figure S4a). RPE is derived from forebrain [61], so neural progenitor-OTX2–7 may be related with the RPE development. Neural progenitor-ERBB3–9 exhibits specific expression of known neural crest (NC) cell markers (*ERBB3*, *SOX9*, and *EDNRA*) [62–64]. So neural progenitor-ERBB3–9 may be related with the NC cell development (Additional file 1: Figure S4a).

Cluster muscle cell consists of four sub-clusters, including muscle-LDHA-3, muscle-FBN2–4, muscle-CRYAB-13, and muscle-GABRP-12. These sub-clusters have specific gene expression pattern and gene expression distribution (Fig. 3c and d and Additional file 1: Figure S3e–h). Muscle-LDHA-3 is enriched for *LDHA*, *POSTN*, and *IGF2* [65]; muscle-FBN2–4 is enriched for *FBN2*, *NCAM1*, and *SERPINE2* [66]; muscle-CRYAB-13 is enriched for *CRYAB*, *COL1A1*, and *LGALS1* [67, 68]; muscle-GABRP-12 is enriched for *GABRP*, *CXCL12*, and *TRIML2* [69]. Combined with the differentiation trajectory, we think these muscle sub-clusters were divided for both different cell types and different differentiation stages (Additional file 1: Figure S4c). Skeletal muscle cell differentiation related genes are enriched in muscle-FBN2–4 and muscle-CRYAB-13; angiogenesis related genes are enriched in muscle-GABRP-12; glycolytic process and insulin receptor signaling pathway related genes are enriched in muscle-LDHA-3 (Additional file 1: Figure S4b). The sub-clusters analysis indicated the diversity of differentiation direction in neural and muscle cell clusters.

### Construction of hPSC early differentiation trajectory

**Fig. 4:**
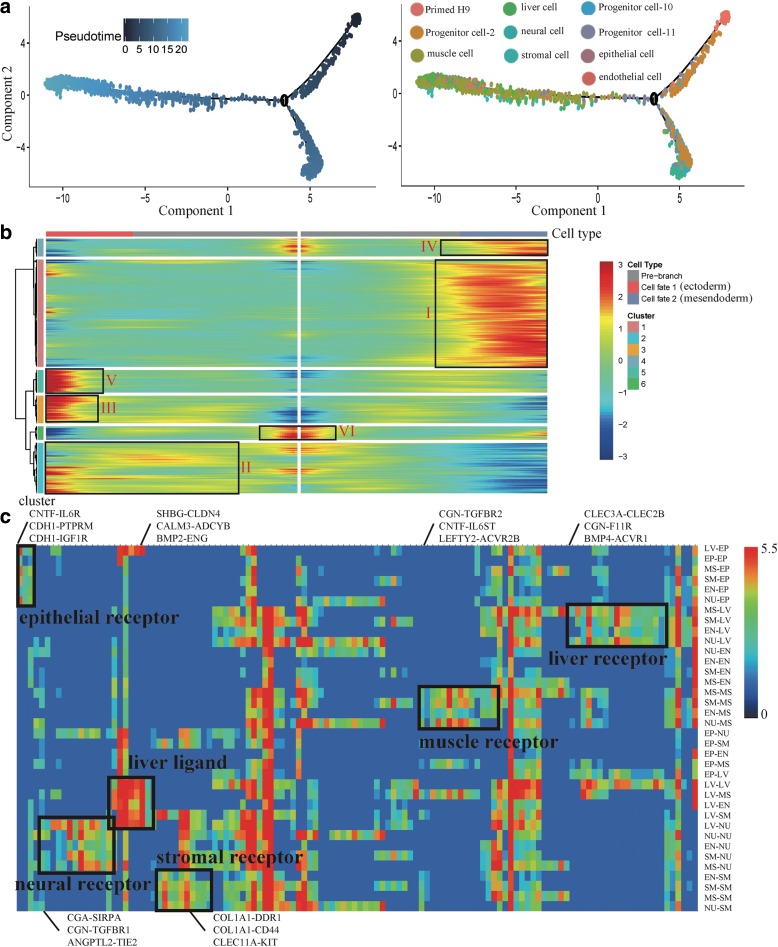
EBs simulate the early development in vivo. **a** Differentiation trajectory of EBs constructed by Monocle. **b** *Heatmap* shows the gene expression dynamics during EB differentiation. Genes (*row*) are clustered and cells (*column*) are ordered according to the pseudotime development. Genes are listed in Additional file 6: Table S5. Gene clusters I–VI were selected for further analysis. **c** *Heatmap* shows the mean number of cell–cell interactions. LV liver cell, EP epithelial cell, MS muscle cell, SM stromal cell, EN endothelial cell, NU neural cell. List of ligand-receptor pairings (*column*) and cell–cell pairings (*row*) are listed in Additional file 7: Table S6

We used Monocle [44] to order single cells through EB differentiation and construct the whole lineage differentiation trajectory with a tree-like structure (Fig. 4a). We found two branches following EB differentiation, including an ectoderm branch and a mesendoderm (mesoderm and endoderm) branch. The ectoderm branch only consists of cells from neural cell cluster. The mesendoderm branch consists of cells from the muscle cell, endothelial cell, stromal cell, liver cell, and epithelial cell clusters. This differentiation trend is similar to the development in vivo that primed epiblasts develop into embryonic ectoderm and primitive streak (embryonic mesoderm and endoderm). It indicates that the differentiation trajectory of whole EBs can simulate the development in vivo. We used a specific heatmap to show the gene expression dynamics of these two branches (Fig. 4b and Additional file 1: Figure S5a). From pre-branch (Primed H9) to cell fate 1 (ectoderm), we found some gene clusters with specific expression pattern, including II (cluster 2), III (cluster 3), and V (cluster 5). We defined the genes cluster VI (cluster 6) and I (cluster 1), which are highly expressed in Primed H9 and cell fate 2 (mesendoderm), respectively. We performed GO enrichment analysis to reveal the different functions of these gene clusters (Additional file 1: Figure S5b). Nervous system development related GO terms are significantly enriched in cluster II, III, and V. In neural differentiation, cells get neural characteristics at the early stage of differentiation. Cluster I is related with kidney development, heart development, skeletal system development, angiogenesis, and lung cell differentiation.

The differentiation trend of EBs is similar to the development in vivo, because 3D EBs have complex cell adhesions and paracrine signaling system, which can establish various interactions among different cell types [70]. Based on the expression of ligands or complementary receptors on every cell, we calculated the number of interactions among different cell types and showed potential cell-cell interactions in a network (Additional file 1: Figure S6) [71]. These ligand-receptor pairings suggest extensive crosstalk among six types of progenitor cells (Fig. 4c). The one-to-many and many-to-one relationships exist between receptors and ligands. For example, liver cell receptor *CLEC2B* can bind ligand *CLEC3A* from muscle cells, stromal cells, endothelial cells, and neural cells; liver ligand *SHBG* can bind to receptor *CLDN4* on all types of cells, which indicate the important roles of liver cells in the differentiation of other cell types. These ligand-receptor pairings may reveal the cell–cell interactions during the development in vivo.

**Fig. 5:**
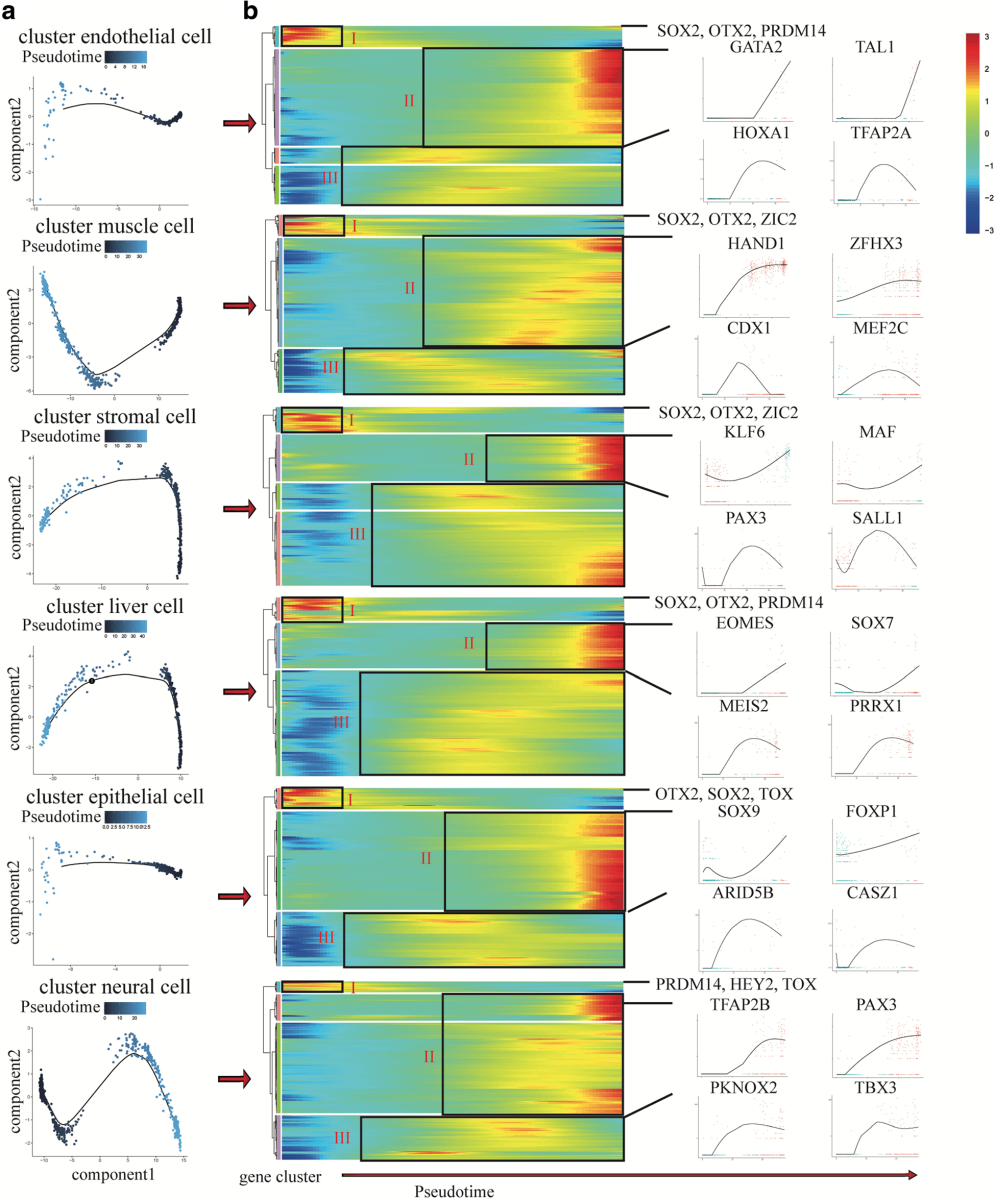
Differentiation trajectories of progenitor cells derived from hPSC. **a** Differentiation trajectories of progenitor cells constructed by Monocle. **b** *Heatmaps* show TFs expression dynamics during differentiation. Genes are listed in Additional file 8: Table S7. Genes (*row*) are clustered and cells (*column*) are ordered according to the pseudotime development. In each heatmap, TFs are divided into three clusters (I, II, and III). Specific TFs are listed on the *right* to show their expression dynamics

The whole lineage differentiation trajectory cannot reveal the single-cell gene expression dynamics of each progenitor cell, so we constructed differentiation trajectory for each cell type with day 8 EBs (Fig. 5a). Transcription factors (TFs) play a key role in the regulation of development and differentiation. Based on the dynamics of their expression patterns, the TFs associated with each differentiation trajectory were divided into three clusters (I, II, III) (Fig. 5b). Cluster I TFs are highly expressed at the initial stage of differentiation. Primed H9, the common starting point of differentiation, highly express cluster I TFs (e.g. *SOX2*, *PRDM14*, and *ZIC2*) [72]. Cluster II TFs are highly expressed at the terminal stage of differentiation, so these TFs indicate the characteristics of each progenitor cell, including cluster endothelial cell (e.g. *GATA2* and *TAL1*) [73], cluster muscle cell (e.g. *HAND1* and *ZFHX3*) [74], cluster stromal cell (e.g. *KLF6* and *MAF*) [75], cluster liver cell (e.g. *EOMES* and *SOX7*) [76], cluster epithelial cell (e.g. *SOX9* and *FOXP1*) [77], and cluster neural cell (e.g. *PAX3* and *TFAP2B*) [78]. Cluster I and II TFs indicate that the start points and end points of our differentiation trajectories are correct. Cluster III TFs are highly expressed at the middle stage of differentiation, including cluster endothelial cell (e.g. *HOXA1* and *TFAP2A*), cluster muscle cell (e.g. *CDX1* and *MEF2C*), cluster stromal cell (e.g. *PAX3* and *SALL1*), cluster liver cell (e.g. *MEIS2* and *PRRX1*), cluster epithelial cell (e.g. *ARID5B* and *CASZ1*), and cluster neural cell (e.g. *PKNOX2* and *TBX3*). These TFs are the candidate genes to control the differentiation of each progenitor cell. We also performed Kyoto Encyclopedia of Genes and Genomes (KEGG) enrichment analysis to reveal the major differential signaling pathways involved in the differentiation, including cluster endothelial cell (e.g. MAPK and Hippo), cluster muscle cell (e.g. Prolactin and Estrogen), cluster stromal cell (e.g. Wnt and Hippo), cluster liver cell (e.g. Prolactin and cGMP-PKG), cluster epithelial cell (e.g. Hippo and AMPK), and cluster neural cell (e.g. Hippo and cGMP-PKG) (Additional file 1: Figure S7). These differentiation trajectories show us the key TFs and signaling pathways that related to the differentiation process and may provide evidences for the optimization of the differentiation system in vitro.

### Construction of naïve hPSC reset trajectory

**Fig. 6:**
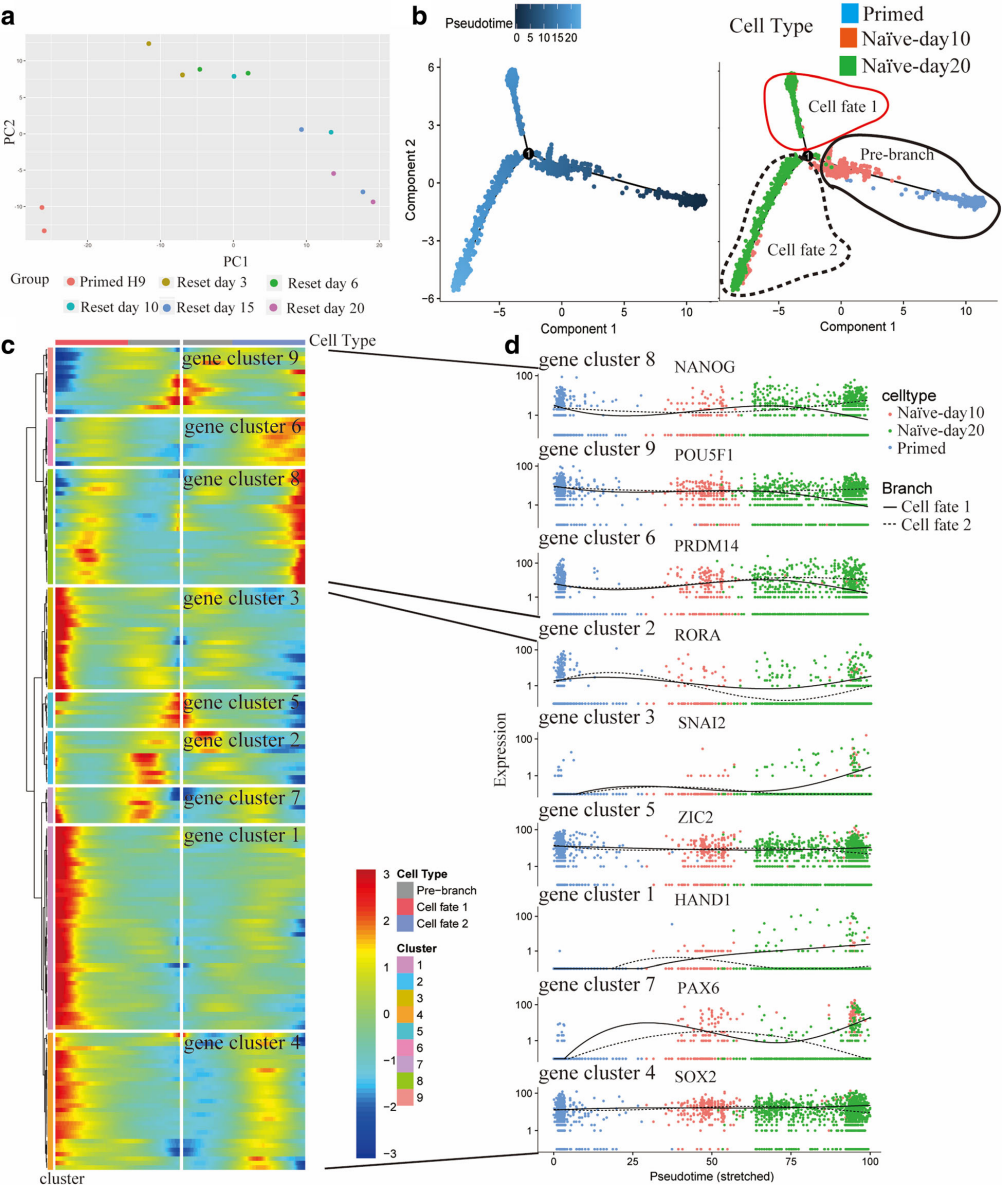
Construction of naïve hPSC reset trajectory by pseudotime analysis. **a** PCA analysis of bulk RNA-seq shows the correlation of hPSCs with different states. Reset H9 was sampled at day 3, day 6, day 10, day 15, and day 20. **b** H9 reset trajectory constructed by Monocle. **c** *Heatmap* shows TFs expression dynamics during the cellular-state transition process. Genes (*row*) are clustered and cells (*column*) are ordered according to the pseudotime development. Genes are listed in Additional file 9: Table S8. **d** TFs expression dynamics. Full line: cell fate 1; Imaginary line: cell fate 2

We reset Primed H9 into Naïve-like H9 by RSeT, a commercial medium based on NHSM formula [10]. Through bulk RNA-seq, we found the state of Naïve-like H9 was stable after 15–20 days domestication in RSeT media (Fig. 6a). We confirmed the naïve state via morphology, immunofluorescence of surface markers and pluripotent transcription factors, quantitative PCR (qPCR) of naïve and primed genes [79, 80], and flow cytometry of surface markers [81] (Additional file 1: Figure S8).

We performed pseudotime analysis to study cell state transition process (from day 0 to day 20) using scRNA-seq data (Fig. 6b). Day 10 RSeT samples are at the intermediate state followed Primed H9. Day 20 RSeT samples are divided into two branches (cell fate 1 and cell fate 2). The RSeT culture process causes the heterogeneity of the Naïve-like H9. The expression pattern shows that only cell fate 2 branch directs to the naïve state with high expression of pluripotent TFs (e.g. *POU5F1*, *NANOG*, and *PRDM14*) (Fig. 6c and d). Cell fate 1 branch directs to a differentiation state with gradual downregulation of pluripotent TFs (e.g. *POU5F1* and *NANOG*) and upregulation of lineage specifier genes (e.g. *HAND1*, *SNAI2*, and *PAX6)*. Though these lineage specifier genes are upregulated at the middle stage in both branches, they are downregulated at the terminal stage of cell fate 2. The differential expression dynamics of lineage specifier genes may help us to understand the reset process.

**Fig. 7:**
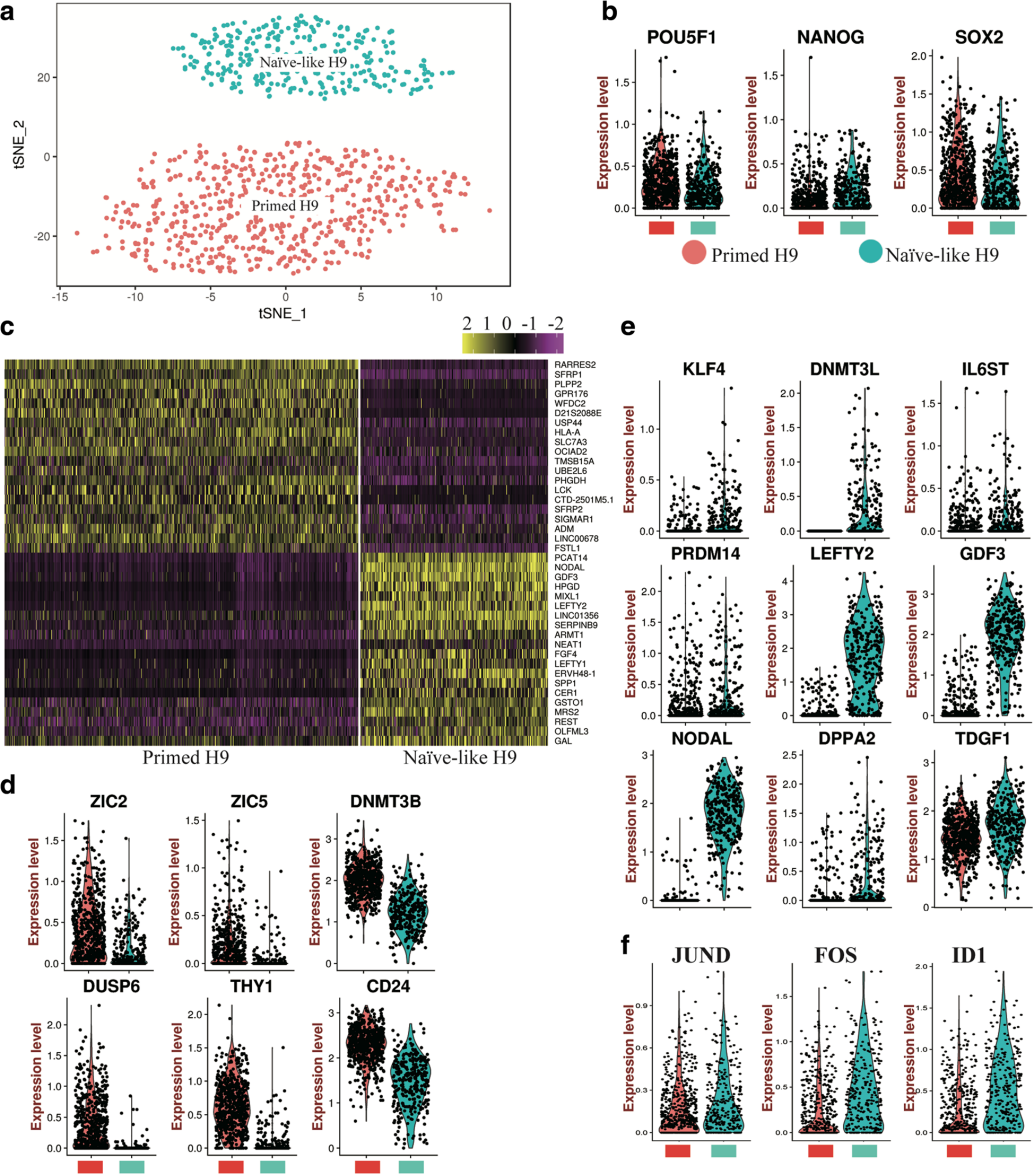
Comparison of Primed and Naïve-like H9 at single-cell level. **a** scRNA-seq t-SNE plot of Primed and Naïve-like H9. Naïve-like H9 was selected from day 20 Reset H9. **b** *Violin plots* show the expression level distributions of pluripotent transcription factors (*POU5F1*, *NANOG*, and *SOX2*). **c** *Heatmap* shows the distinct gene expression pattern of Primed and Naïve-like H9. Top 20 differential genes are shown. Genes used are listed in Additional file 10: Table S9. **d**–**f** *Violin plots* show the expression level distributions of primed genes (**d**), naïve genes (**e**), and MAPK related genes (**f**)

We extracted cell fate 2 branch as Naïve-like H9 to compare gene expression pattern between Naïve-like and Primed H9 at single-cell level (Fig. 7a). Though expression distributions of pluripotent TFs (e.g. *POU5F1*, *NANOG*, and *SOX2*) are similar (Fig. 7b and Additional file 1: Figure S9a), there are significant differences in gene expression signatures between Naïve-like and Primed H9 (Fig. 7c). Primed genes (e.g. *ZIC2*, *ZIC5*, *DNMT3B*, *DUSP6*, *THY1*, and *CD24*) are enriched in Primed H9 (Fig. 7d). *NODAL*, *LEFTY2*, *GDF3*, *KLF4*, *DNMT3L*, *IL6ST*, *PRDM14*, *DPPA2*, and *TDGF1* are highly expressed in Naïve-like H9 as previously reported (Fig. 7e) [79, 80]. These results confirmed the “naïve” state of our Naïve-like H9. These gene expression characteristics also exist in Naïve-like H1 (Additional file 1: Figure S10a–c and S10f). We performed surface marker analysis to show the candidate surface genes, which can distinguish naïve hPSCs from primed hPSCs (Additional file 1: Figure S9b). The surface markers of Primed H9 include *CUZD1*, *KCNQ2*, *CLDN10*, *PODXL*, etc.; the surface markers of Naïve-like H9 include *CNTNAP2*, *FZD5*, etc.

**Fig. 8:**
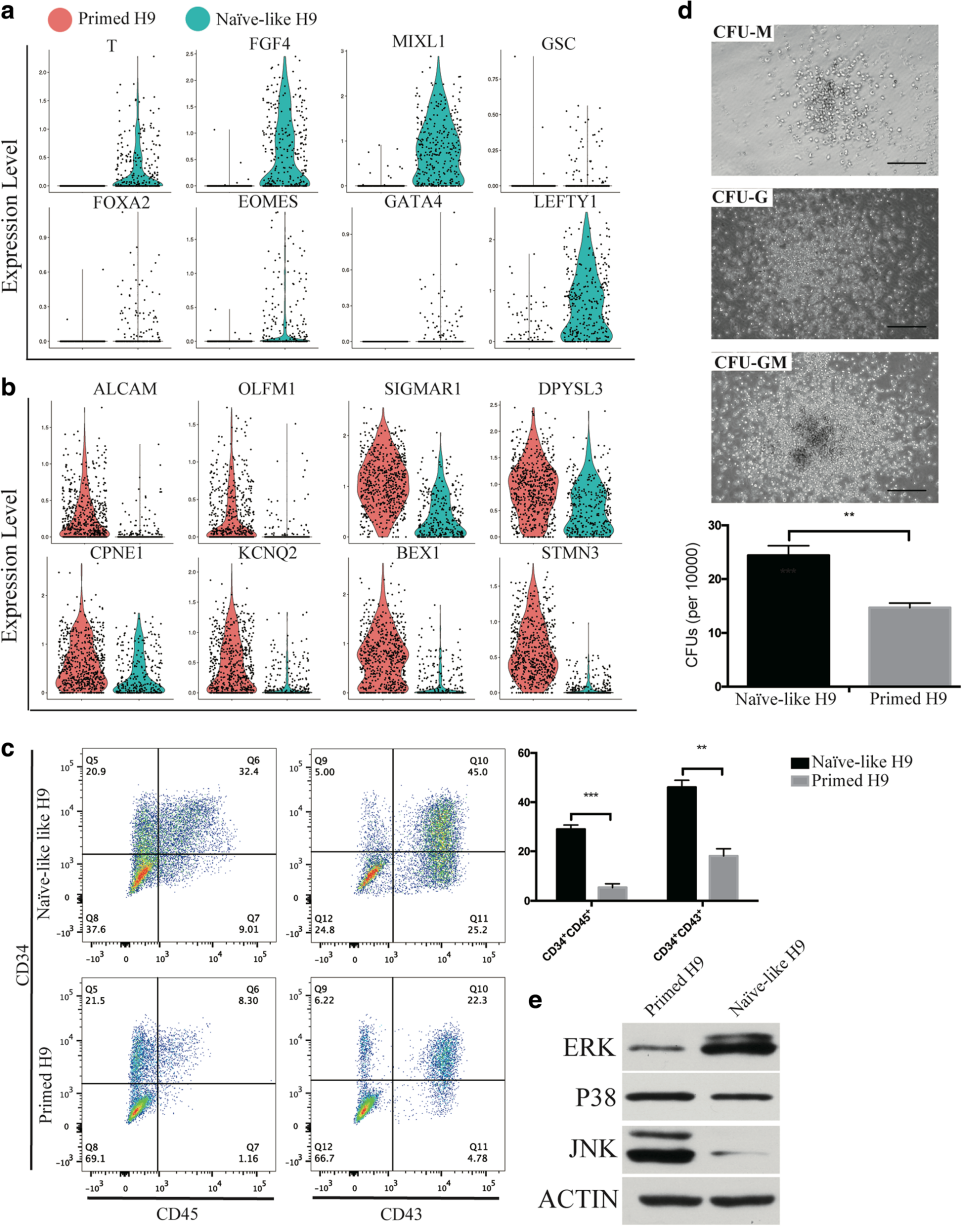
Hematopoietic differentiation bias of Naïve-like H9. **a**, **b** *Violin plots* show the expression level distributions of mesendoderm genes (*T*, *FGF4*, *MIXL*, *GSC*, *FOXA2*, *EOMES*, *GATA4*, and *LEFTY1*) (**a**) and neural genes (*ALCAM*, *OLFM1*, *SIGMAR1*, DPYSL3, *CPNE1*, *KCNQ2*, *BEX1*, and *STMN3*) (**b**). **c** Flow cytometry analysis of hematopoietic progenitors derived from hPSCs. Significant difference was assessed by the t-test. ***p < 0.001, **p < 0.01, *p < 0.05. **d** The morphology and number of hematopoietic CFUs. Scale bars = 100 μm. **e** Western blot analysis of MAPK (ERK1/2, JNK, and P38) in naïve and primed H9

We performed GO enrichment analysis and established GO term diagram of Naïve-like and Primed H9 (Additional file 1: Figure S9c). Primed H9 have high expression of major histocompatibility complex I related genes as previously reported [72]. And genes related with nervous system (ectoderm) development are also enriched in Primed H9. In Naïve-like H9, genes related with gastrulation and endoderm development and MAPK signaling pathway are enriched. GO term diagram of Naïve-like H1 also shows MAPK signaling pathway enrichment (Additional file 1: Figure S10 g). Interestingly, MAPK signaling pathway is also enriched in the differentiation trajectory of endothelial cell cluster, which derives from mesendoderm (Additional file 1: Figure S7). We checked the expression distribution of germ layer genes in both H9 and H1. Genes related with mesendoderm development (*T*, *FGF4*, *MIXL*, *LEFTY1*, *EOMES*, etc. [40]) are highly expressed in Naïve-like hPSCs (Fig. 8a and Additional file 1: Figure S10e); genes related with nervous development (*ALCAM* [82], *OLFM1* [83], *SIGMAR1* [84], etc.) are highly expressed in Primed hPSCs (Fig. 8b and Additional file 1: Figure S10d). We suspected that Naïve-like hPSCs have the differentiation bias to tissue cells related with endothelial-hematopoietic lineages [85, 86]. We used hematopoietic differentiation system to compare the differentiation ability between Naïve-like and Primed H9. The percentage of CD34^+^CD45^+^ cells and CD34^+^CD43^+^ cells are higher in Naïve-like H9, which generates more colony-forming units (CFUs) than Primed H9 as well (Fig. 8c and d). It suggests that Naïve-like H9 has better potency for hematopoietic differentiation. We checked the protein level of MAPK (p38, JNK, and ERK1/2) through western blot, and only the ERK1/2 is highly detected in Naïve-like H9 (Fig. 8e), which is consistent with the single cell transcriptome analysis. The high expression of *FOS* (the substrates of MAPK-ERK1/2) [87] and *ID1* (the downstream target of MAPK-ERK1/2) are also detected in Naïve-like H9 (Fig. 7f) [88], which do not affect the pluripotency of Naïve-like H9 (Fig. 7b and Additional file 1: Figure S8). *FOS* can induce the expression of hematopoietic genes [89]. Furthermore, *ID1* is a helix-loop-helix inhibitor and may promote the hematopoietic differentiation [90] like the helix-loop-helix inhibitor TAL1 does [91].

## Discussion

**Fig. 9:**
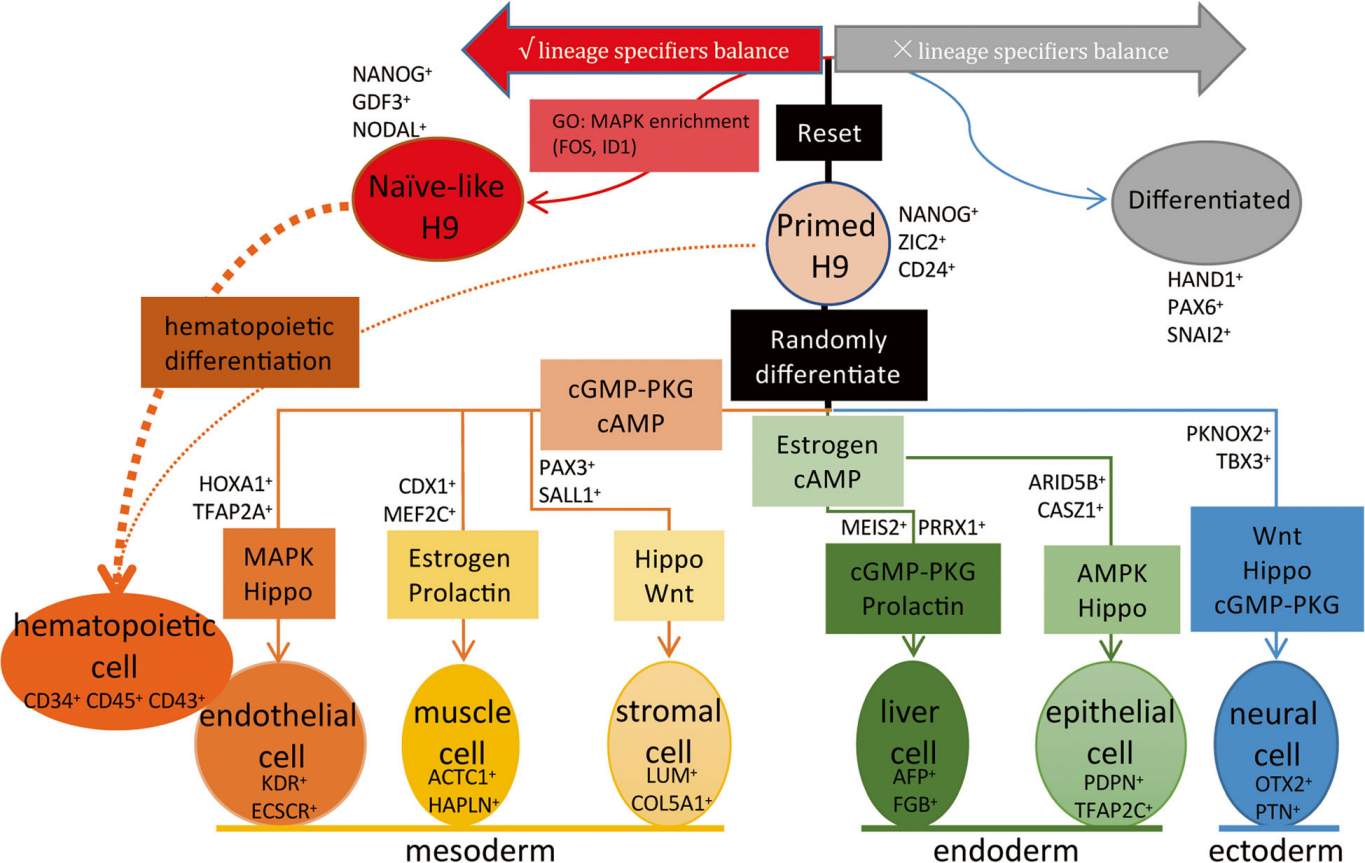
*Snapshot* of scRNA-seq profiling on progenitor cells and hPSCs. Differentiation trajectories of six progenitor cells derived from Primed H9 show key signaling pathways and TFs involved in the differentiation. The balance of lineage specifiers decides the reset result of Primed H9. MAPK-ERK1/2 signaling pathway related genes are enriched in Naïve-like H9, which may contribute to the hematopoietic differentiation bias

In this study, we performed the analysis of scRNA-seq data from a total of 4822 single cells generated from EBs and hPSCs (Fig. 9). Through constructing the early differentiation trajectories of various progenitors identified in EBs, we revealed the key TFs and signaling pathways that direct the differentiation of distinct cell types. Moreover, we constructed the cell–cell interaction network of these cell types and indicated the key roles of liver cells in the differentiation of other cell types. We further reprogramed Primed H9 into Naïve-like H9 to study the cellular-state transition process. We found that genes related with MAPK-ERK1/2 signaling pathway are enriched in endothelial-hematopoietic development and Naïve-like H9. Functionally, Naïve-like H9 show higher potency for differentiation into the hematopoietic lineages. These results provide valuable information for the optimization of differentiation protocols.

The scRNA-seq platform we used is Fluidigm C1 system with the HT IFCs [26]. The old version of IFCs can only capture 96 cells at most [40, 92], so cell sorting or other enrichment strategies are usually performed before scRNA-seq for the recovery of rare cell types. However, HT IFCs can efficiently analyze thousands of single cells without prior enrichment from heterogeneous systems such as EB differentiation (Fig. 2a).

In contrast to monolayer differentiation, EB differentiation system provides 3D structure to establish complex cell adhesions and paracrine signaling, which promote the differentiation and morphogenesis similar to the native tissue development [70]. The interactions between different cell types are important for the development and differentiation. Liver is the essential site for the early hematopoietic development in the embryo stage [85]. Cardiomyocytes and endothelial cell were reported important for the differentiation of liver in EB differentiation [21]. We used the random EB differentiation system to generate various tissue cells of three germ lineages for our hPSC early differentiation trajectory construction (Fig. 9). We found complex interactions among different cell types (Fig. 4c and Additional file 1: Figure S6). Interestingly, liver cells build specific interactions with other cell types using specific ligands and receptors in EBs. The functions of these interactions should be verified in further studies. It may indicate the important role of liver cells in the differentiation of other cell types.

We used the scRNA-seq to study the reset trajectory of Naïve-like H9. In the tree-like trajectory, we found two branches, one directs to success and the other directs to failure (Fig. 6b). After comparing gene expression dynamics, we revealed the cell state transition process, from Primed to Naïve-like H9. We found that some lineage specifier genes (*PAX6*, *HAND1*, et al.) are upregulated at the middle stage (Fig. 6d). In the success branch, those lineage specifier genes are downregulated before the terminal stage. However, in the failure branch, the upregulation is persistent, which lead to differentiation but not the naïve state. The balance of lineage specifier genes can keep the pluripotency of stem cell [93]. We therefore suspected that in the cell state transition process Primed H9 is reprogramed to a pluripotent intermediate state with the balance of lineage specifiers (Fig. 9). When this balance is broken, the intermediate state cells lose their pluripotency and differentiate. Understanding the mechanisms that control the balances of these lineage specifier genes may help us to regulate the pluripotency of hPSCs and optimize differentiation protocols.

Differentiation bias of different hPSCs might be harnessed for better lineage differentiation protocols. Here, we found better potency of hematopoietic differentiation in Naïve-like H9. MAPK-ERK1/2 related genes are highly expressed in Naïve-like H9 but not in Primed H9 (Fig. 7f and Fig. 8e and Additional file 1: Figure S9c). We therefore suspect that MAPK-ERK1/2 contributes to the hematopoietic differentiation bias of Naïve-like H9 (Fig. 9). Though LIF is the key cytokine to keep the “naïve” state of hPSCs [10], it can also activate the MAPK-ERK1/2 signaling pathway [94], which is involved in the differentiation of hPSCs towards endothelial lineages (Additional file 1: Figure S7), and hematopoietic development [87]. The commercial naïve medium RSeT contain the MAPK-ERK1/2 inhibitor (such as PD0325901 [10]). The inhibitor may lead to perturbation of MAPK-ERK1/2 pathway. When the inhibitor is removed and differentiation media are added, hemogenic fate is enhanced for Naïve-like hPSC culture.

## Conclusion

In this study, we used scRNA-seq to map the early differentiation of hPSCs. We identified various lineage-specific progenitor cells and constructed the differentiation trajectories by pseudotime analysis. The gene expression dynamics offer new insights into molecular pathways of early embryonic lineages that can be harnessed for optimization of differentiation protocols.

## Methods

### Cell culture and differentiation

H9 and H1 human ES cells were maintained in mTeSR™1 media (STEMCELL Technologies) on tissue culture plates coated with Matrigel (BD Bioscience) routinely [95]. H9 and H1 were reset into a naïve-like state by RSeT™ media (STEMCELL Technologies) following the instruction [81, 96]. We generated EBs by clone suspension. EBs were differentiated in DMEM/F12 (GIBCO) supplemented with 20% FBS (GIBCO), 50 U/mL penicillin/streptomycin (GIBCO), 2 mM L-Glutamine (GIBCO), 1 × non-essential amino acids, and 100 μM ß-mercaptoethanol (Sigma). In brief, H9 was digested using 0.5 mg/mL Dispase (Invitrogen) for 30 min. Then cell clumps suspended in differentiation media were seeded into an Ultra-Low attachment 6 well plate (Corning). After four days or eight days of culture, EBs were harvested and digested into single-cell suspension in 3 × 10^5^ cells/mL using TrypLE (GIBCO). We did not use cell sorting or other enrichment strategies before single-cell capture. The hematopoietic differentiation of hPSCs was performed using STEMdiff Hematopoietic Kit (STEMCELL Technologies) following the instructions. At day 12, cells were analyzed with flow cytometry. CD34^+^ cells were enriched with EasySep™ CD34 positive selection kit (StemCell Technologies) for CFU assays.

### Colony-forming unit (CFU) assays

CFU assays were performed with MethoCult™ H4034 Optimum methylcellulose-based media (StemCell Technologies) following manufacturer’s instructions. In brief, 3 mL MethoCult™ media with 1 × 10^4^/mL CD34^+^ cells and penicillin-streptomycin were added into each 35 mm low adherent plastic dish. Colonies were counted and identified after 10–14 days of incubation.

### Flow cytometry analysis of cell phenotype

Cells suspended in 100 μL of PBS were incubated with antibodies at 4 °C for 30 min. The samples were measured on BD Fortessa and analyzed by FlowJo software (Tree Star). Antibodies used in our study were listed: anti-Human CD34 (BioLegend, Pacific Blue, clone 581), anti-human CD34 (BioLegend, PE, clone 581), anti-human CD201 (BioLegend, APC, clone RCR-401), anti-Human CD43 (BioLegend, APC, clone 10G7), anti-Human CD45 (BioLegend, FITC, clone HI30), anti-Human CD90 (BD Pharmingen, APC, clone 5E10), and CD24 (BioLegend, PE, clone ML5).

### Immunofluorescence staining and confocal image analysis

Cells were seeded into glass-bottom culture dishes (NEST, 35/15 mm) coated with Matrigel. Cultured cells were fixed in 4% paraformaldehyde at room temperature for 30 min. Then permeabilized treatment was performed at room temperature for 30 min with PBS + 0.2% TritonX-100. Cells were blocked with PBS + 1% BSA + 4% FBS + 0.4% TritonX-100 at room temperature for 1 h. Then cells were incubated with primary antibodies, diluted in PBS + 0.2% BSA + 0.1% TritonX-100, at 4 °C overnight. Cells were incubated with AlexaFluor secondary antibodies (Invitrogen) for 1 h at room temperature. Then cells were incubated with DAPI for 5 min at room temperature. After the second round of fixation, cells were ready for imaging. Olympus IX81-FV1000 was used to collect immunofluorescence images and FV10-ASW 2.1 Viewer was used to process images. The primary antibodies used in our study were listed in Additional file 2: Table S1.

### Western blot analysis

Whole-cell protein were isolated from Primed H9 and Naïve-like H9. Protein samples were incubated with the following primary antibodies in 5%BSA: anti-ERK (Servicebio, Wuhan, China, GB13003–1), anti-JNK (Epitomics, 3496-s), anti-P38 (ABCAM, ab31828), and anti-β-actin (Servicebio, Wuhan, China, GB13001–1). Secondary antibodies were HRP-linked goat anti-mouse, goat anti-rabbit (Servicebio, Wuhan, China, GB23303). Blots were developed using ECL (Servicebio, Wuhan, China, G2014). The primary antibodies used in our study were listed in Additional file 2: Table S1.

### Reverse transcription (RT) and qPCR analysis

Total RNA prepared with EasyPure RNA Kit (Transgen) was reverse transcribed into complementary DNA (cDNA) by TransScript All-in-One First-Strand cDNA Synthesis SuperMix for qPCR kit (Transgen). The diluted cDNA was used as temples in qPCR (ChamQ SYBR qPCR Master Mix-Q311 (Vazyme)). The qPCR platform we used was LightCycler 480 (Roche) and data were analyzed by the ∆∆Ct method. The primers used in our study were listed in Additional file 3: Table S2, including the reference gene (*ACTB*).

### Single-cell capture and scRNA-seq library preparation

We used Fluidigm C1 system and C1 high-throughput integrated fluidics circuits (HT IFCs) to perform the single-cells capture and library construction as instruction described. A total of 4000–8000 cells were loaded onto a medium-sized (10–17 μm) HT IFCs. The efficiency of capture was measured under the microscope. The capture sites without cell or with more than one cell were marked and excluded from further analysis. C1 system captured and converted all polyadenylated messenger RNA (mRNA) into cDNA with the cell-specific barcodes. After reverse transcription and preamplification, cDNA was prepared as samples for next-generation sequencing using library tagmentation and 3’end enrichment. Samples harvested from HT IFCs were used to create libraries for Illumina sequencing with Illumina Nextera XT DNA Library kit.

### Bulk RNA-seq library construction

We used mRNA Capture Beads (VAHTS mRNA-seq v2 Library Prep Kit for Illumina, Vazyme) to extract mRNA from total RNA. PrimeScript™ Double Strand cDNA Synthesis Kit (TaKaRa) was used to synthesize double-stranded cDNA from purified polyadenylated mRNA templates. We used TruePrep DNA Library Prep Kit V2 for Illumina (TaKaRa) to prepare cDNA libraries for Illumina sequencing.

### Sequencing data analysis

The sequenced reads were mapped against the reference GRCh38 using STAR v2.5.2a [97]. scRNA-seq expression data, quantified by counts via featureCounts v1.5.1 [98], were analyzed with Seurat v2.0.1 (PCA, Cluster, t-SNE and cluster) [43]. In brief, the Seurat object was generated from digital gene expression matrices. The parameter of “Filtercells” is nGene (2000 to 8800) and transcripts (-Inf to 6e + 05). In the standard pre-processing workflow of Seurat, we selected 8706 variable genes for following PCA. Then we performed cell cluster and t-SNE. Fifteen principal components were used in cell cluster with the resolution parameter set at 1.5. Marker genes of each cell cluster were outputted for GO and KEGG analysis, which were used to define the cell types. Cell clusters were annotated with the information of cell types and germ layers. Digital gene expression matrices with annotations from Seurat were analyzed by Monocle v2.3.6 (pseudotime analysis) [44]. TFs from AnimalTFDB [99] and surface genes [100] were used to filter the gene lists. The cell–cell interactions were constructed by igraph v1.12 as previously reported [71]. The count of cell–cell interactions was based on the ligands-receptors pairings [101]. We used DAVID [55] to perform GO and KEGG analysis. GO terms were visualized by REVIGO [102] and Cytoscape [103]. Bulk RNA-seq data, quantified by FPKM via RSEM v0.4.6 [104], were analyzed with DEseq2 v1.14.1 [105].

## Additional files

Additional file 1: **Figure S1.** Quality control of the dataset. **Figure S2.** Surface marker analysis and GO enrichment analysis of lineage progenitors. **Figure S3.** FeaturePlot of specific genes from neural and muscle sub-clusters. **Figure S4.** Differentiation trajectories and GO analysis of neural and muscle sub-clusters. **Figure S5.** GO analysis and expression dynamics of gene clusters I–VI. **Figure S6.** Network of potential cell–cell interactions in EBs. **Figure S7.** Signaling pathways involved in differentiation of various progenitor cells. **Figure S8.** The identification of Naïve-like H9. **Figure S9.** Surface marker analysis and GO analysis of Primed and Naïve-like H9. **Figure S10.** The identification and GO analysis of Primed and Naïve-like H1 (Additional file 11: Table S10, Additional file 12: Table S11, Additional file 13: Table S12, Additional file 14: Table S13, Additional file 15: Table S14). (DOCX 19207 kb)

Additional file 2: **Table S1.** Immunofluorescence antibody. (XLSX 33 kb)

Additional file 3: **Table S2.** qPCR primer. (XLSX 47 kb)

Additional file 4: **Table S3.** List of genes used in Fig. 2b for heatmap. (XLSX 468 kb)

Additional file 5: **Table S4.** List of genes used in Fig. 3a and c for heatmap. (XLSX 99 kb)

Additional file 6: **Table S5.** List of genes used in Fig. 4b for heatmap. (XLSX 38 kb)

Additional file 7: **Table S6.** List of ligand-receptor pairs and cell–cell pairs used in Fig. 4c for heatmap. (XLSX 12 kb)

Additional file 8: **Table S7.** List of genes used in Fig. 5b for heatmaps. (XLSX 43 kb)

Additional file 9: **Table S8.** List of genes used in Fig. 6c for heatmap. (XLSX 12 kb)

Additional file 10: **Table S9.** List of genes used in Fig. 7c for heatmap. (XLSX 44 kb)

Additional file 11: **Table S10.** List of GO terms used in Additional file 1: Figure S2. (XLSX 64 kb)

Additional file 12: **Table S11.** List of GO terms used in Additional file 1: Figure S4. (XLSX 73 kb)

Additional file 13: **Table S12.** List of GO terms used in Additional file 1: Figure S5. (XLSX 56 kb)

Additional file 14: **Table S13.** List of signaling pathways used in Additional file 1: Figure S7a. (XLSX 20 kb)

Additional file 15: **Table S14.** List of GO terms used in Additional file 1: Figure S9. (XLSX 22 kb)
