# Supplementary material for: Mapping human pluripotent stem cell differentiation pathways using high throughput single-cell RNA-sequencing
Source: Genome Biol. 2018 Apr 5;19:47. doi: 10.1186/s13059-018-1426-0 (PMC5887227; doi:10.1186/s13059-018-1426-0)
Supplement: Supplementary file 1 — Figure S1. Quality control of the dataset. Figure S2. Surface marker analysis and GO enrichment analysis of lineage progenitors. Figure S3. FeaturePlot of specific genes from neural and muscle sub-clusters. Figure S4. Differentiation trajectories and GO analysis of neural and muscle sub-clusters. Figure S5. GO analysis and expression dynamics of gene clusters I–VI. Figure S6. Network of potential cell–cell interactions in EBs. Figure S7. Signaling pathways involved in differentiation of various progenitor cells. Figure S8. The identification of Naïve-like H9. Figure S9. Surface marker analysis and GO analysis of Primed and Naïve-like H9. Figure S10. The identification and GO analysis of Primed and Naïve-like H1 (Additional file 11: Table S10, Additional file 12: Table S11, Additional file 13: Table S12, Additional file 14: Table S13, Additional file 15: Table S14). (DOCX 19207 kb) [file 13059_2018_1426_MOESM1_ESM.docx]

**Additional file 1**

**
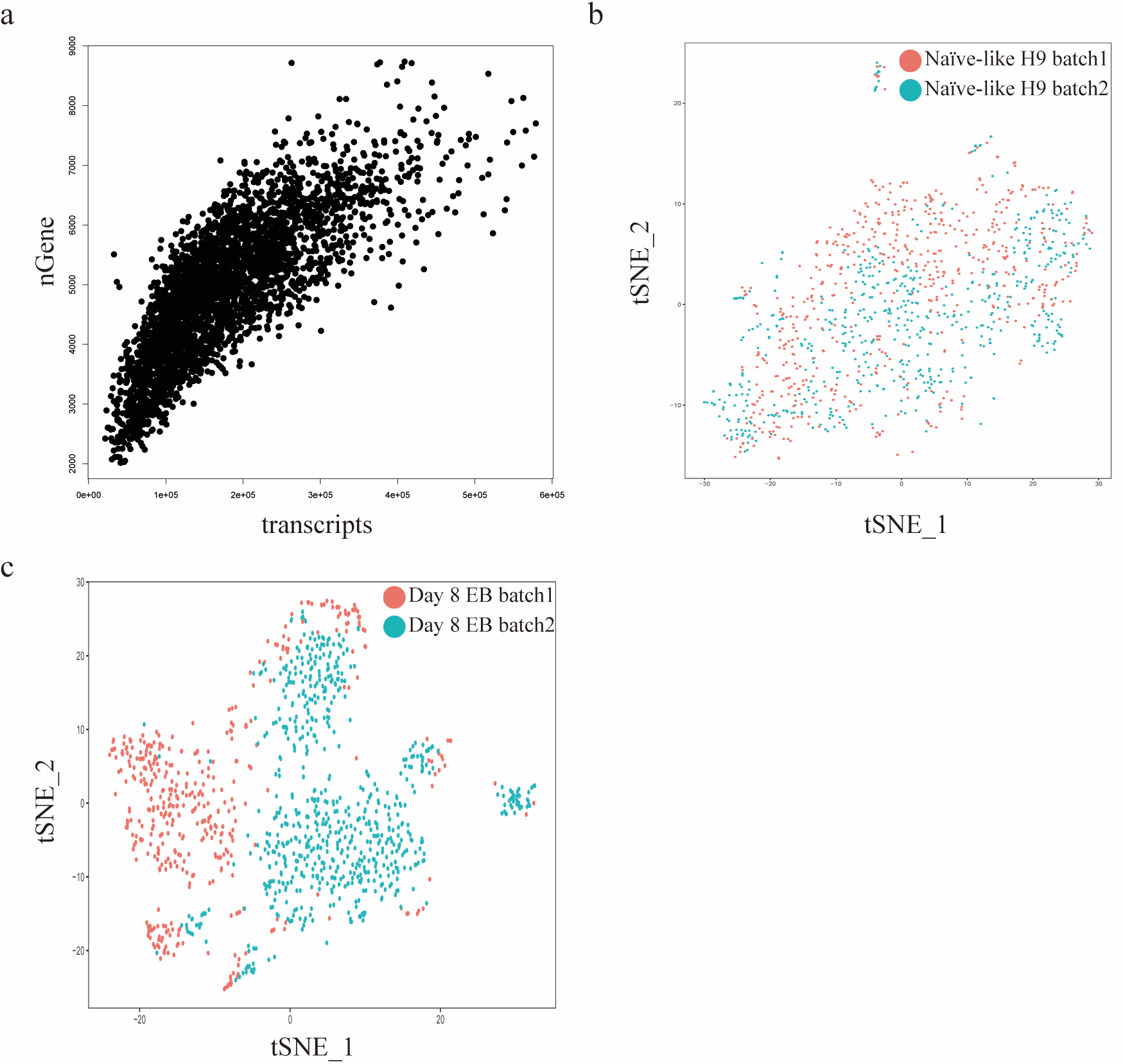
**

**Fig. S1 Quality control of the data set. a** Scatter diagram showed the gene numbers (nGene) and transcript numbers of single cells after cell filtering. **b-c** Batch information of Naïve-like H9 (b) and day 8 EBs (c) mapped on t-SNE plots.

**
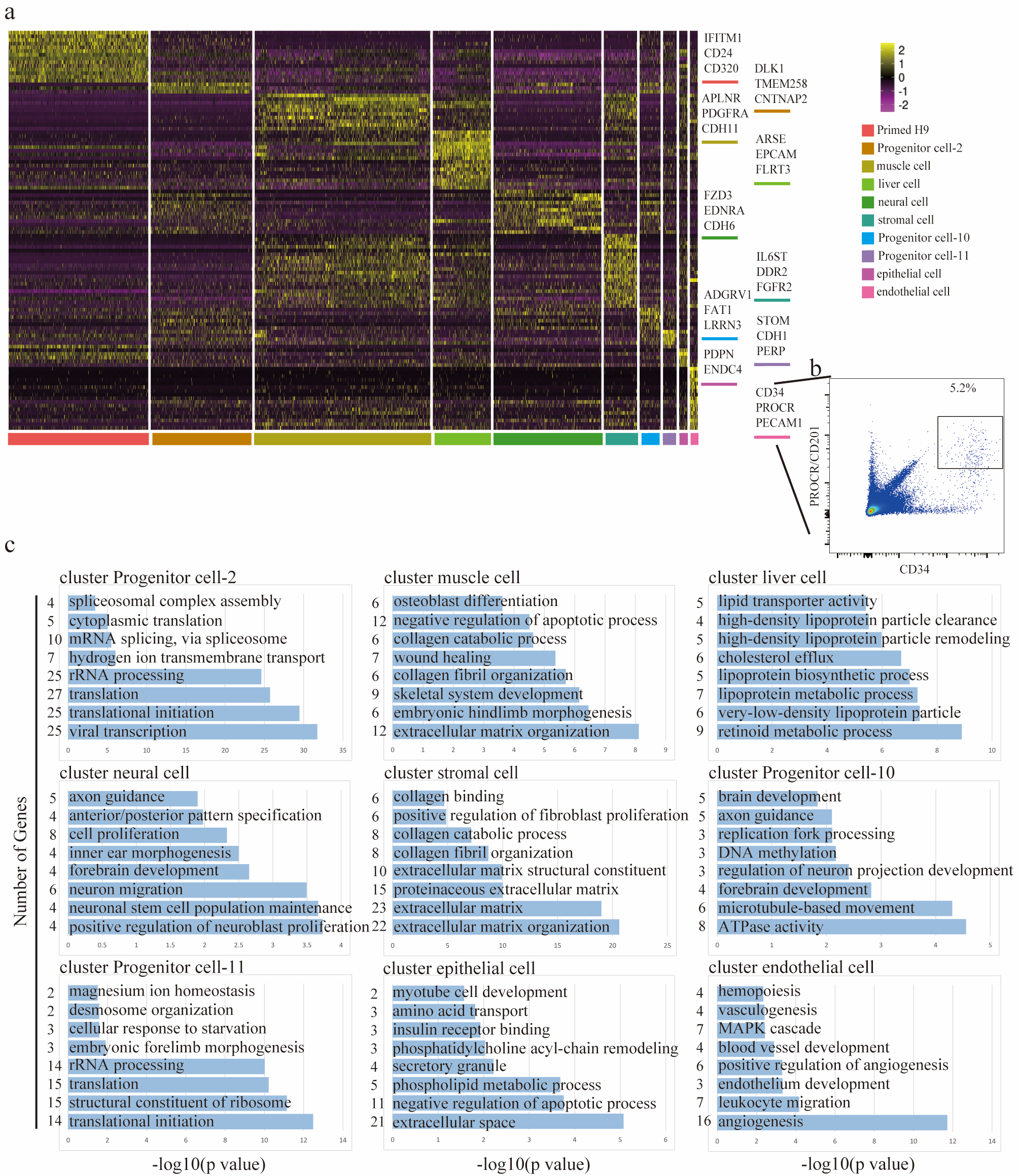
**

**Fig. S2 Surface marker analysis and GO enrichment analysis of lineage progenitors. a** Heatmap shows the specific surface markers of each progenitor cell. **b** Flow cytometry showed the percentage of CD34^+^CD201^+^ endothelial cells in EBs. **c** Top 100 genes of each cell type were selected to perform the GO analysis. Eight GO terms are shown with *p*<0.05. Gene number of each GO term is listed on the left. *p* value is shown as –log(*p value*). GO terms used are listed in Additional file 11: Table S10.

**
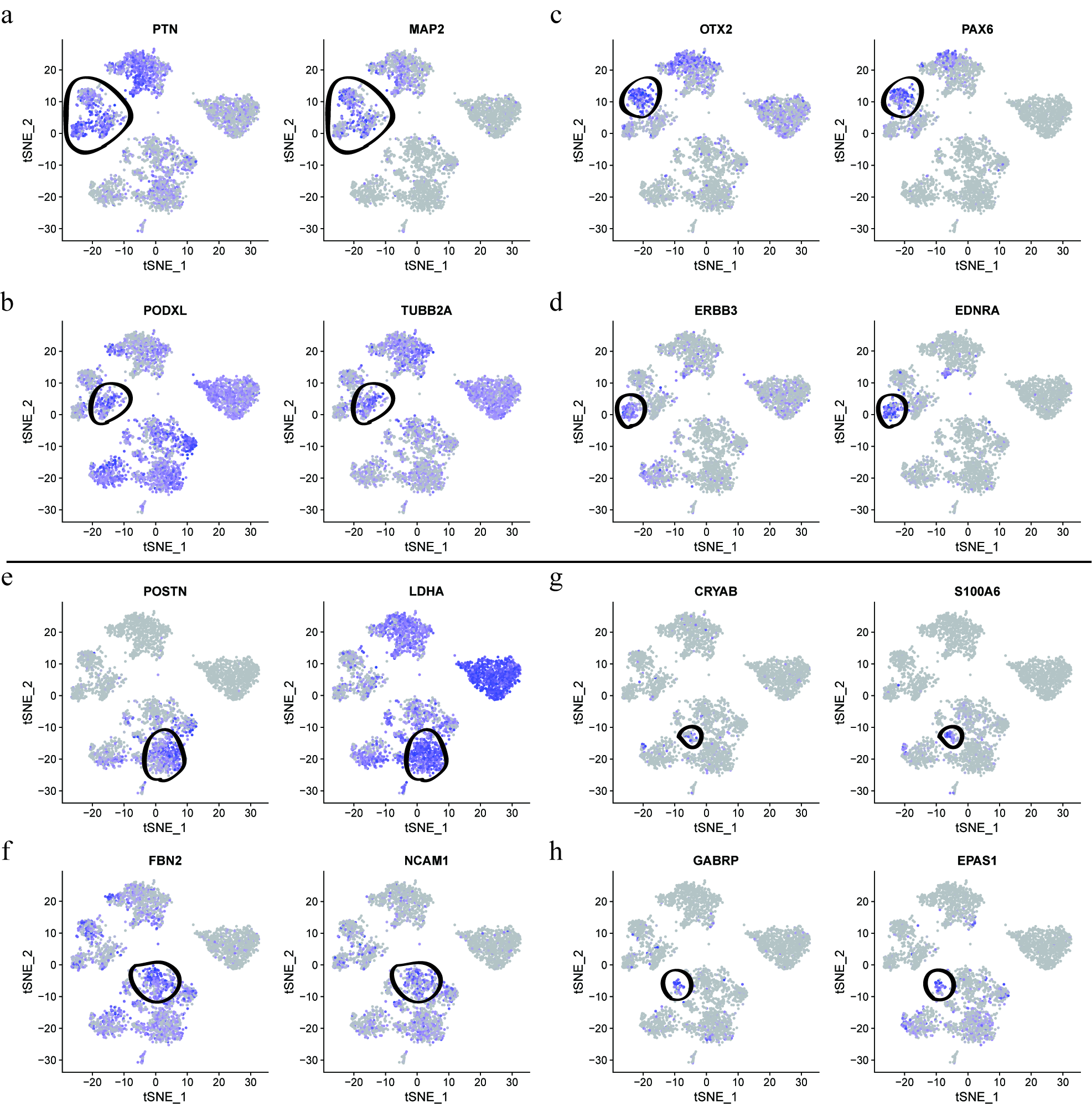
**

**Fig. S3 FeaturePlot of specific genes from neural and muscle sub-clusters.** **a-d** Genes enriched in cluster neural cell (a), neural progenitor-PODXL-6 (b), neural progenitor-OTX2-7 (c), and neural progenitor-ERBB3-9 (d). **e-h** Genes enriched in cluster muscle-LDHA-3 (e), muscle-FBN2-4 (f), muscle-CRYAB-13 (g), and muscle-GABRP-12 (h) . Black circles mark the sub-clusters.


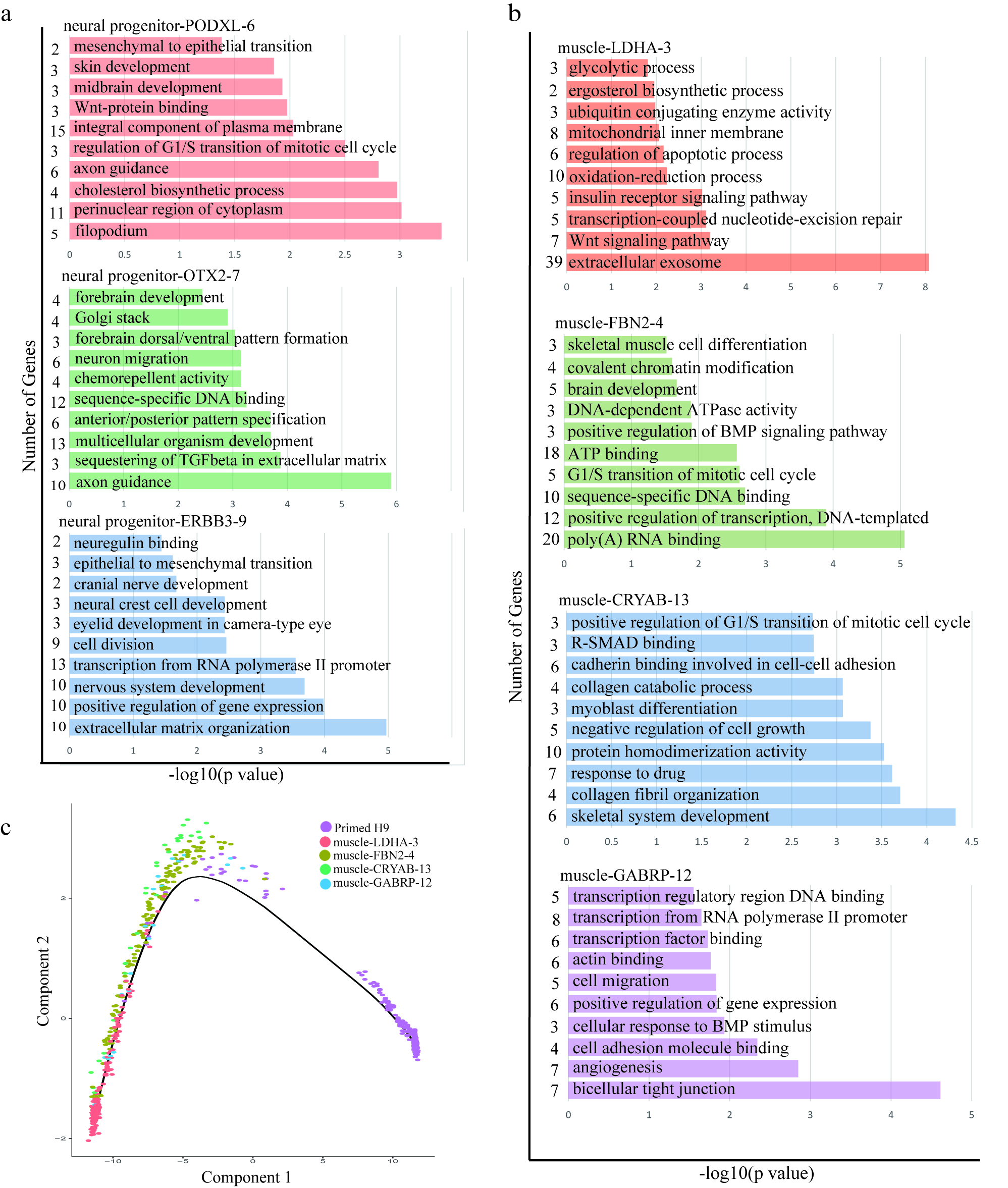


**Fig. S4 Differentiation trajectories and GO analysis of neural and muscle sub-clusters. a-b** Ten GO terms of each neural sub-cluster (a) and muscle sub-cluster (b). Top 100 genes of each sub-cluster were selected to perform the GO enrichment analysis. Gene number of each GO term is listed on the left. *p*<0.05. *p* value was shown as –log(*p* value). GO terms used are listed in Additional file 12: Table S11. **c** Differentiation trajectories of muscle sub-clusters constructed by Monocle.

**
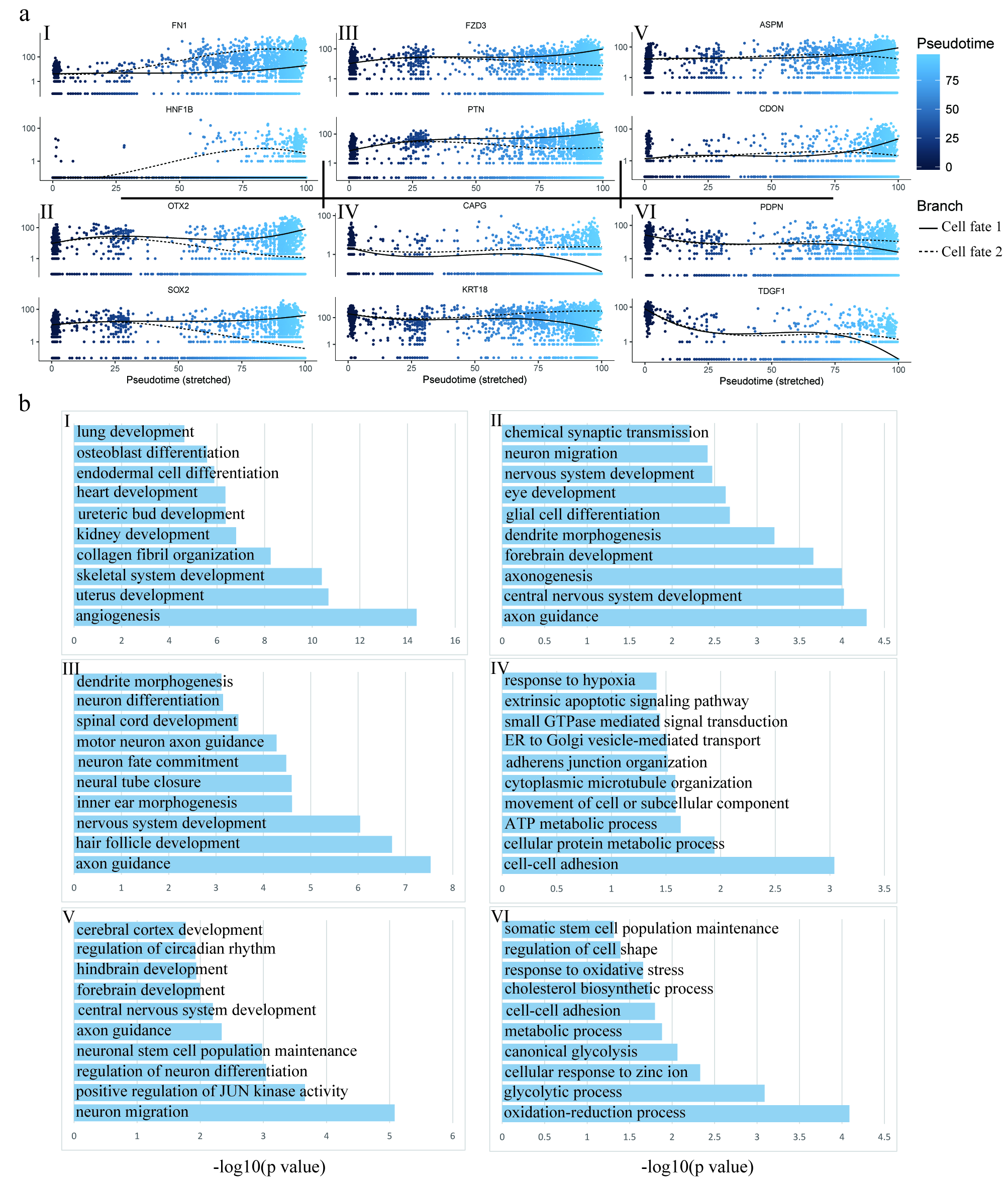
**

**Fig. S5 GO analysis and expression dynamics of gene clusters I-VI.** **a** Gene expression dynamics. Full line: cell fate 1; Imaginary line: cell fate 2. **b** Ten GO terms were shown with *p*<0.05. *p* value was shown as –log(*p* value). GO terms used are listed in Additional file 13: Table S12.


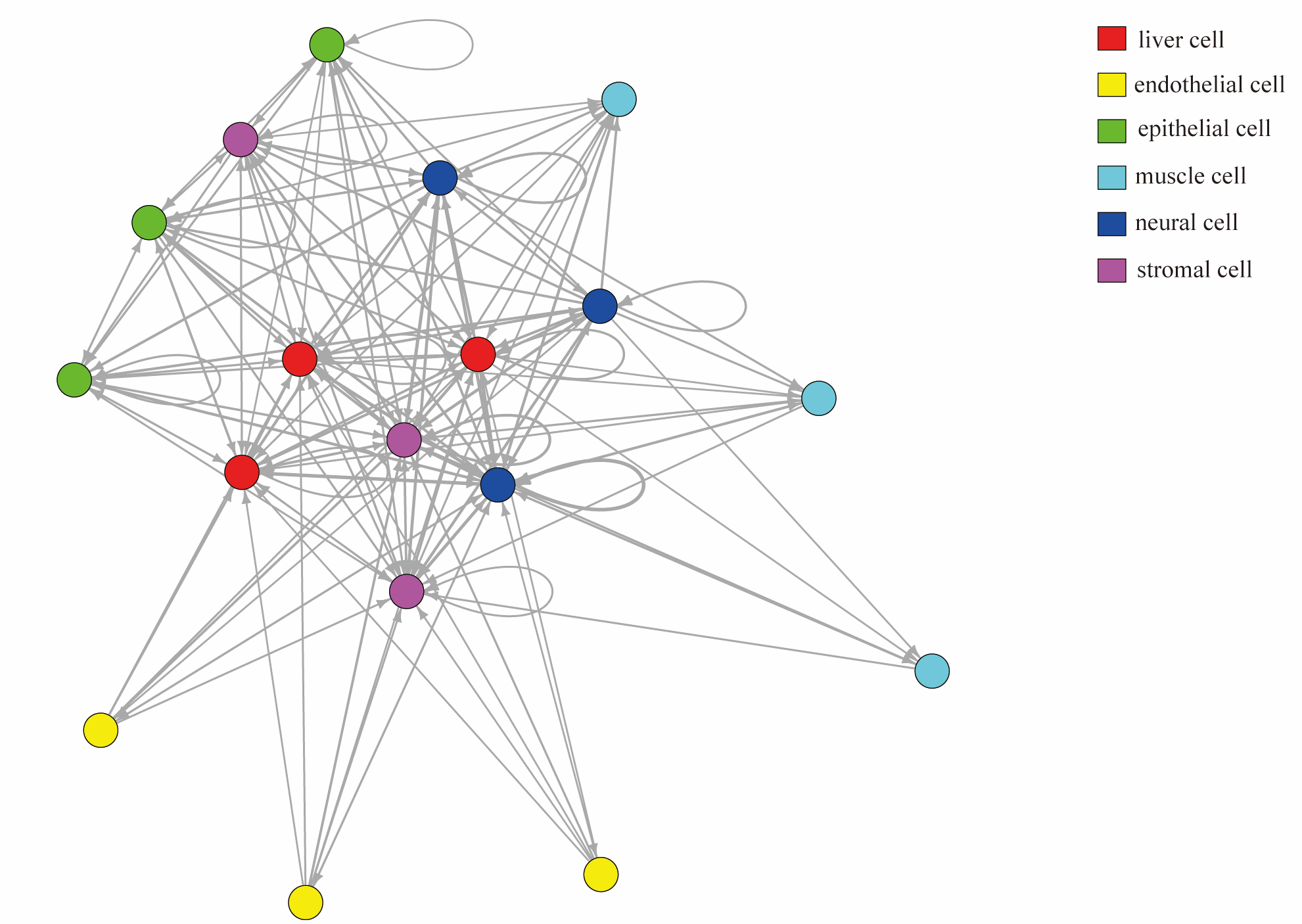


**Fig. S6 Network of potential cell–cell interactions in EBs.** Nodes: cell types; thickness of arrow-line: the sum of ligand-receptor pairings.

**
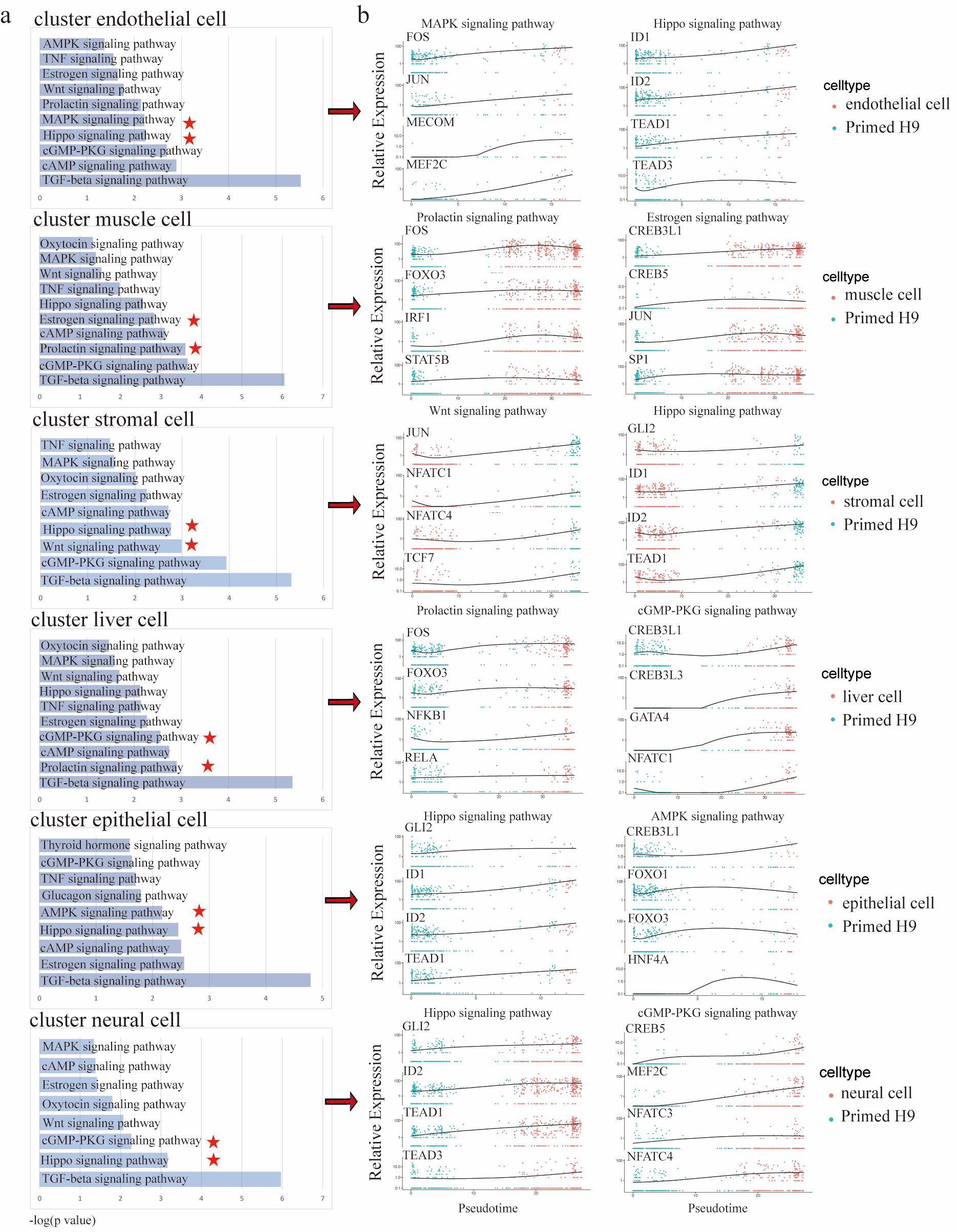
**

**Fig. S7 Signaling pathways involved in differentiation of various progenitor cells. a** KEGG pathway analysis of six progenitor cells using II and III gene clusters (from Fig. 5b). Signaling pathways used are listed in Additional file 14: Table S13. Ten signaling pathways are shown with *p*<0.05. *p* value is shown as –log(*p* value). The differential signaling pathways have star marks. **b** TFs expression dynamics of differential signaling pathways.

**
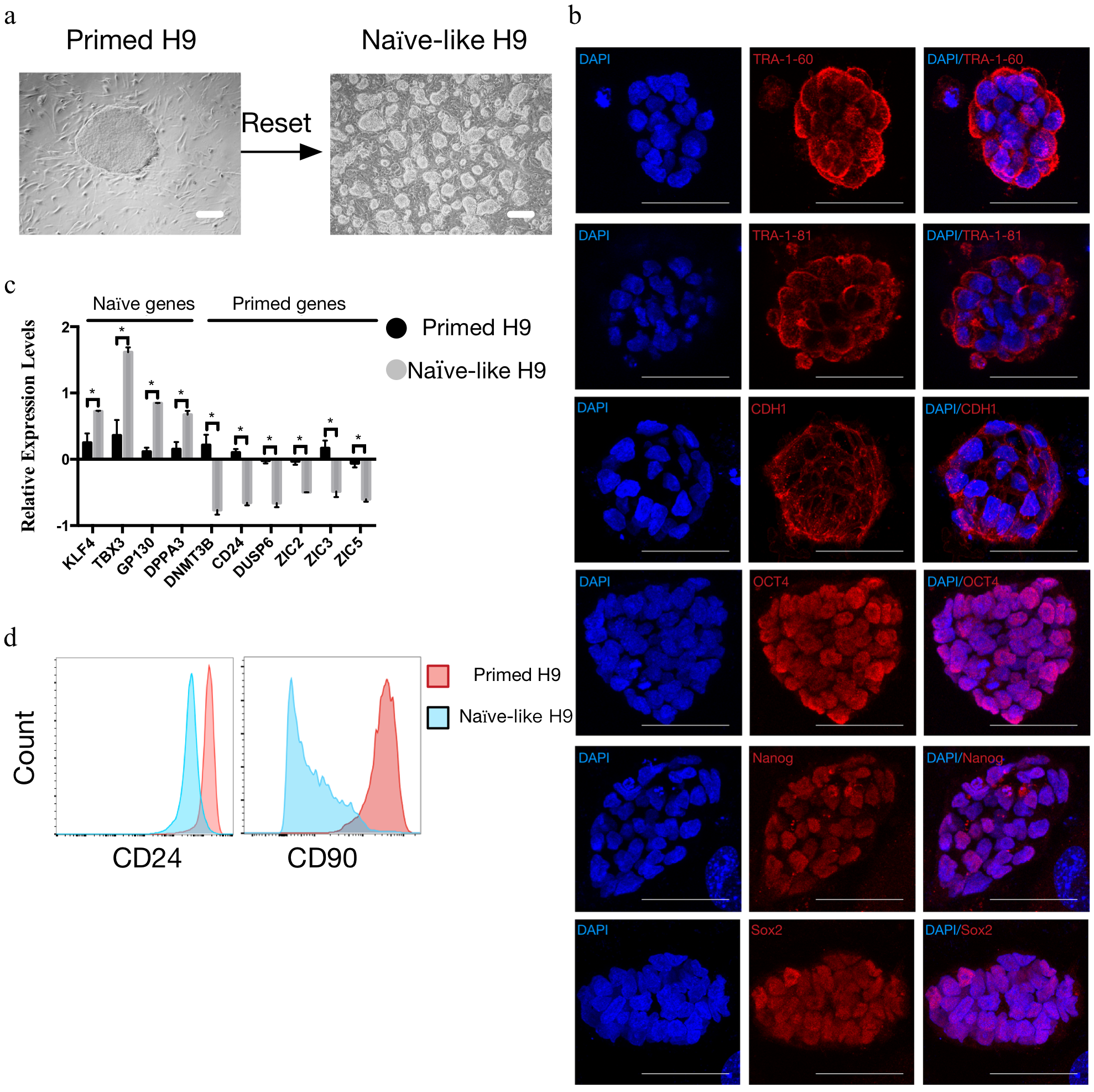
**

**Fig. S8 The identification of Naïve-like H9. a** The morphological change of H9 from primed to the naïve state. **b** Immunofluorescence staining of pluripotent markers, *Oct4*, *Nanog*, *Sox2*, *CDH1*, *TRA*-1-81 and *TRA*-1-60. Scale bars=100μm. **c** qPCR analysis of specific genes in Primed and Naïve-like H9. Significant difference was assessed by the t-test. *p*＜0.05. **d** Flow cytometer analysis of *CD24* and *CD90* in Primed and Naïve-like H9.


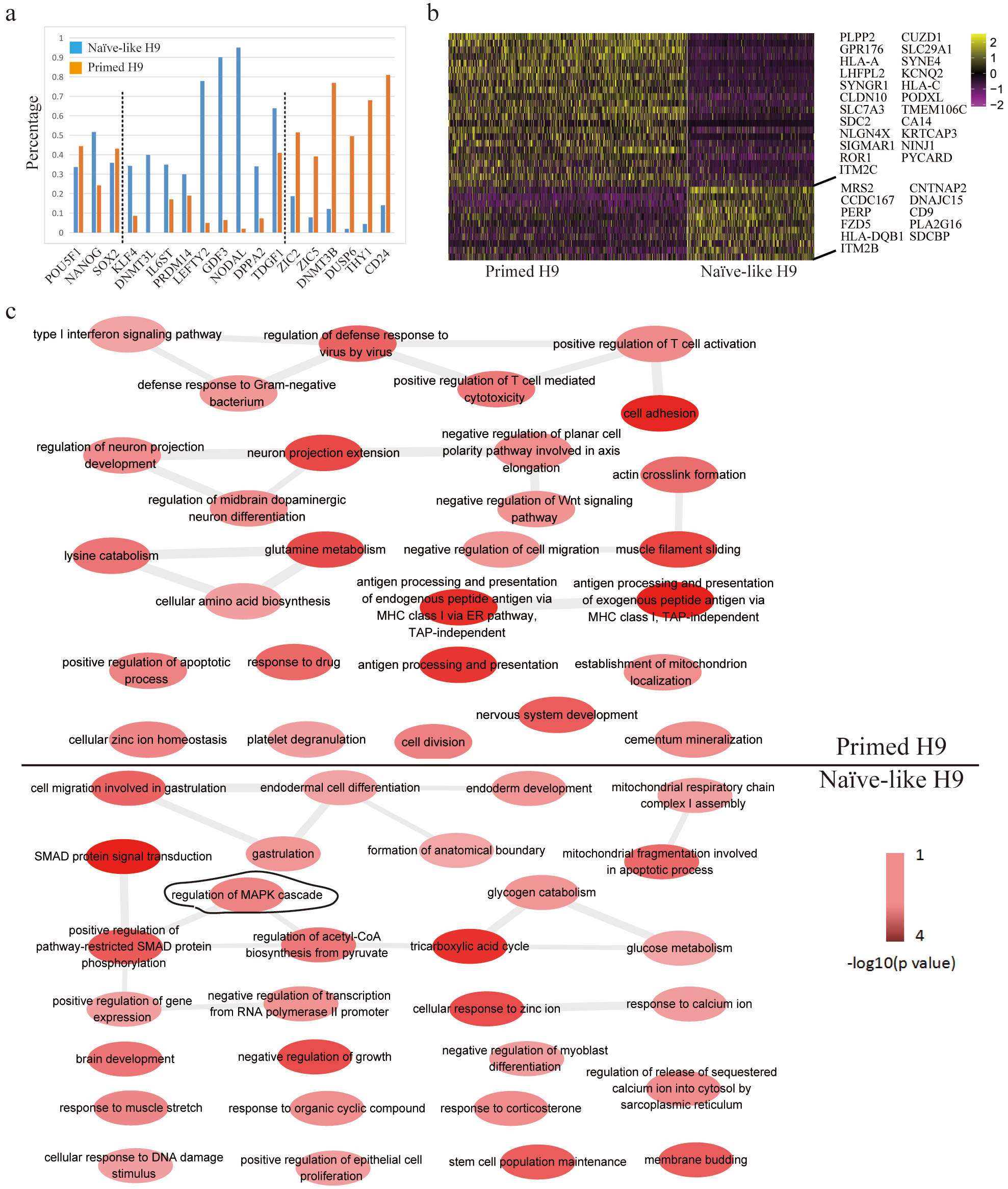


**Fig. S9 Surface marker analysis and GO analysis of Primed and Naïve-like H9. a** The percentage of genes (naïve genes, primed genes and pan-pluripotency genes) in Naïve-like H9 and Primed H9. The base line to judge a gene expression is the average of gene expression. **b** Heatmap shows the specific surface markers of Primed and Naïve-like H9. **c** GO terms of Primed and Naïve-like H9 were visualized by REVIGO and Cytoscape. Color indicates the *p* value of GO terms. GO terms used are listed in Additional file 15: Table S14.

**
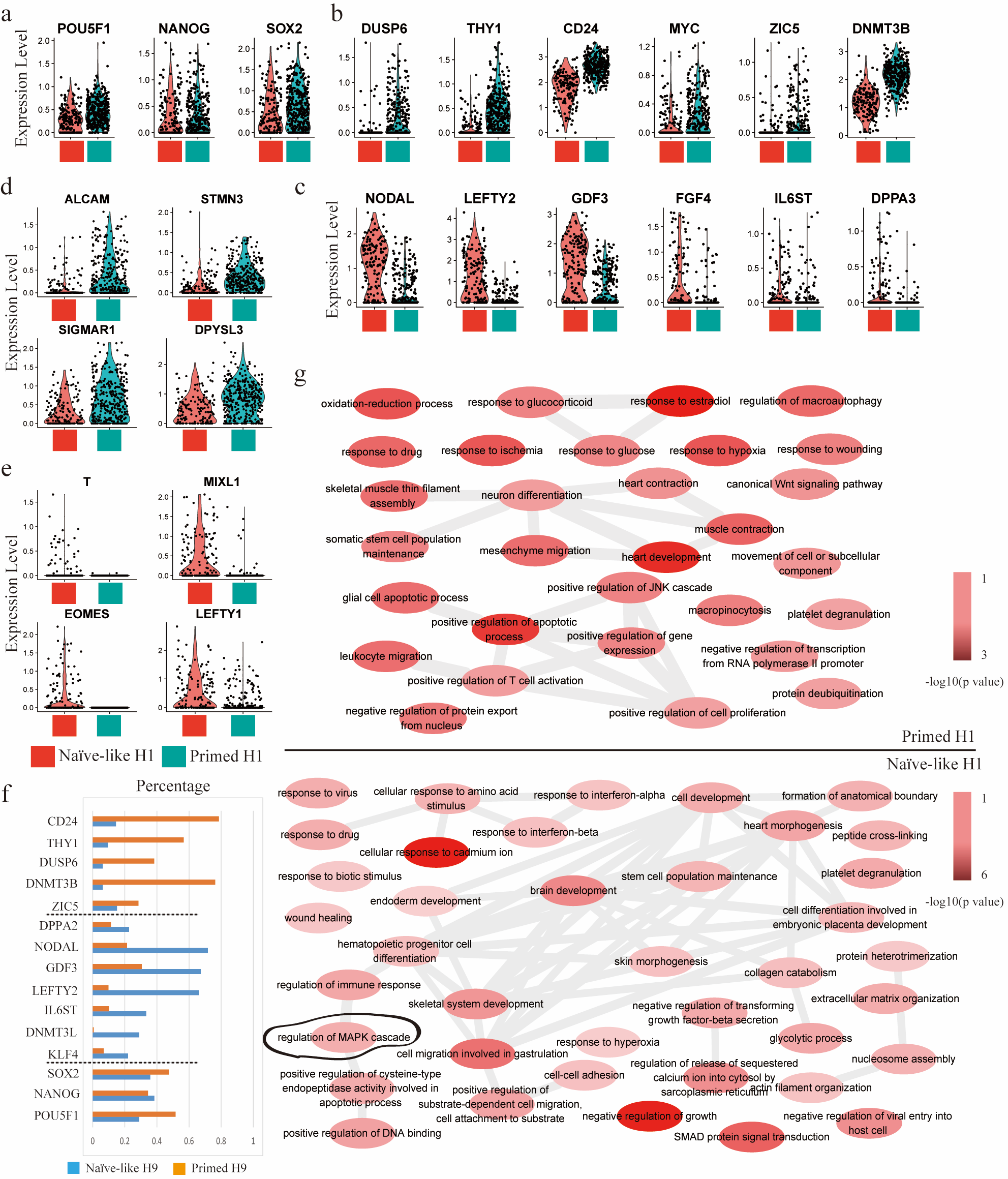
**

**Fig. S10 The identification and GO analysis of Primed and Naïve-like H1. a-e** Violin plots show the expression level distributions of pluripotent transcription factors (POU5F1, NANOG and SOX2) (a), primed genes (b), naïve genes (c), neural genes (d), and mesendoderm genes (e). **f** The percentage of genes (naïve genes, primed genes and pan-pluripotency genes) in Naïve-like H1 and Primed H1. The base line to judge a gene expression is the average of gene expression. **g** GO terms of Primed and Naïve-like H1 were visualized by REVIGO and Cytoscape. Color indicates the *p* value of GO terms.
